# Analysis on agricultural carbon emissions transfer of inter-provincial trade in China

**DOI:** 10.1371/journal.pone.0311744

**Published:** 2024-11-21

**Authors:** Xidong Zhang, Juan Zhang, Wenhao Fu, Ziting Pei, Bin Zhang, Mufan Zhuang

**Affiliations:** 1 Institute of Ecology and Sustainable Development, Shanghai Academy of Social Sciences, Shanghai, China; 2 College of Philosophy, Law & Political Science, Shanghai Normal University, Shanghai, China; 3 School of Mathematical Sciences, Suzhou University of Science and Technology, Suzhou, China; 4 School of Business, Suzhou University of Science and Technology, Suzhou, China; 5 Research Center of Rural Economy, Ministry of Agriculture and Rural Affairs of the People’s Republic of China, Beijing, China; Sichuan Agricultural University, CHINA

## Abstract

The "carbon peaking and carbon neutrality goals" has put forward new requirements for China’s agricultural carbon emission reduction. It is easy to ignore the carbon emission transfer caused by agricultural trade if the reduction responsibility of carbon emission is merely defined from the supply side. Therefore, it is necessary to conduct in-depth research on agricultural carbon transfer for reasonably dividing the responsibility of agricultural carbon reduction in different provinces. In this study, the cross-section data of 31 provincial-level administrative regions in China in 2015, 2018 and 2021 were used to calculate the agricultural carbon emissions of each province from the production side, and the agricultural carbon transfer model was applied to study the spatial transfer characteristics of agricultural carbon emissions. The results show that: (1) In 2015, 2018, and 2021, the net carbon transfer in Chinese agriculture was 125.76 million tons, 132.49 million tons, and 133.02 million tons, respectively, accounting for 11.97%, 13.31%, and 13.61% of agricultural carbon emissions respectively. (2) The net input area of agricultural carbon emissions formed a spatial distribution pattern of four major regions which are concentrated in the southeast coastal areas, and the gap of net input of emissions was narrowing among the regions. Shanghai, Zhejiang, and Fujian are the regions with the largest net agricultural carbon input among the net input regions. The net agricultural carbon input increased from 43.00 million tons in 2015 to 52.71 million tons in 2021. In Guangdong and Guangxi, agricultural carbon emissions decreased from 41.34 million tons in 2015 to 35.61 million tons in 2021. In Sichuan, Chongqing, and Guizhou, agricultural carbon emissions decreased from 22.98 million tons in 2015 to 14.20 million tons in 2021. Beijing and Tianjin are the regions with the smallest net agricultural carbon input among the four net input regions, with the net agricultural carbon input increasing from 12.53 million tons in 2015 to 13.92 million tons in 2021. (3) The net output area of agricultural carbon emissions also formed a spatial distribution pattern of four major regions, and they were concentrated in the north of China with the center of gravity of net output shifting to the north. In 2015, Heilongjiang and Jilin were the regions with the largest net carbon output among the four net output regions. The net agricultural carbon output increased from 38.45 million tons in 2015 to 39.44 million tons in 2021. In Xinjiang and Gansu, the net agricultural carbon output increased from 15.87 million tons in 2015 to 23.37 million tons in 2021. In Inner Mongolia, the net agricultural carbon output increased from 17.03 million tons in 2015 to 23.05 million tons in 2021. Henan and Anhui have consistently maintained a high level of net agricultural carbon output, the net agricultural carbon output decreased from 35.54 million tons in 2015 to 25.68 million tons in 2021. On the whole, the spatial transfer of agricultural carbon emissions in China shows the characteristics of "north carbon transport to south" bounded by the Yangtze River. This paper believes that agricultural policies of carbon emission reduction should be formulated at both ends of agricultural supply and demand due to the spatial transfer of agricultural carbon emissions, which is not only conducive to stabilizing the production enthusiasm of major agricultural production provinces, but also conducive to controlling carbon emissions in output and input regions. For this purpose, the study puts forward countermeasures and suggestions to promote the reduction of agricultural carbon emission in different provinces, so as to better leverage the green and low-carbon development in the agricultural field under the guidance of the "carbon peaking and carbon neutrality goals".

## Introduction

Agriculture is an important source of global greenhouse gas emissions [1], and 19%-29% of global greenhouse gas emissions come from food production systems [2]. According to the Fourth National Communication on Climate Change of the People’s Republic of China, the agricultural carbon emissions in 2017 accounted for 6.4% of the total national carbon emissions. As the largest agricultural country in the world, China emits 11%-12% of the world’s agricultural carbon emissions [3]. Promoting green and low-carbon development in agriculture is one of the important ways to achieve the dual-carbon goal [4, 5]. However, the production and consumption of agricultural products in China are becoming more and more spatially separated [6] due to spatial differences in natural resource endowments [7], imbalance in resource utilization and distribution [8], and continuous flow and transfer of population [9]. Therefore, there have been disagreement among provinces in sharing the reduction responsibility of carbon emissions [10]. Inter-regional agricultural trade can connect production and consumption areas, and it is an effective way to allocate resources through market means [11]. However, trade among regions with different resource endowments will have impact on resource and environment [12], and such impact can shift from the consumption space to the production space [13, 14]. Therefore, the conduction of carbon transfer calculation and the research on temporal spatial characteristics of inter-provincial agricultural trade will be helpful to identify and divide agricultural reduction responsibilities of carbon emission with important reference value for developing scientific and reasonable inter-provincial agricultural reduction strategies of carbon emission based on shared responsibilities.

In the study of carbon emissions transfer, many scholars have carried out research on the implied carbon transfer in international trade, aiming to explore the carbon leakage between developed and developing countries [15–17]. An earlier study on the carbon footprint of EU member states found that the EU transfers environmental stress to other parts of the world by importing carbon-intensive products [18]. Further studies have shown that industrialized countries transfer high-emission and high-pollution industries to developing countries led by China [19] and result in the phenomenon of "carbon leakage" [20] due to the constrains of emission reduction commitments. However, since the financial crisis, carbon emissions in developing countries have declined, especially in China [21]. Studies at the national level have concluded that developed countries have higher carbon emissions measured by consumption, while developing countries have higher carbon emissions measured by production, and there is a large carbon imbalance implied by trade between developing countries and developed ones [22]. In developed countries, the increase of carbon emissions on the consumption side is much higher than the reduction of carbon emissions on the production side, and the problem of carbon leakage cannot be ignored [23].

With the continuous improvement of inter-provincial input-output data in China, the measurement of carbon emissions in China’s regional production side and consumption side has gradually attracted scholars’ attention. In China’s regional supply and demand chain, carbon emissions driven by final consumption and export have both increased significantly [24], and the scale of carbon emission transfer caused by inter-provincial trade is generally larger than that caused by foreign trade [25]. Within China, consumption in relatively developed coastal areas has a higher measured carbon emissions [26, 27], the final demand led to the increase of carbon emissions in central and western regions [28]. In coastal and inland regions, eastern and western regions, the carbon emissions caused by consumption and production present a non-equilibrium feature and carbon emissions have shifted spatially [29–31].

In general, although domestic and foreign scholars have conducted extensive research on the issue of carbon transfer implied by trade, few scholars have paid attention to the issue of carbon transfer implied by agricultural trade. Most studies on agricultural trade focus on virtual water and virtual arable land flow caused by grain trade [32–36]. In addition, existing studies on agricultural carbon emissions transfer mostly focus on the carbon transfer implied by food trade, pointing out that food trade increases the total carbon emissions [37], and carbon emissions from food trade presents a spatial flow pattern of "carbon transport from North to south and from central to west". Carbon emissions from grain trade show the characteristics of large "north-south" flow and small "east-west" flow [38]. On this basis, some scholars also studied the inter-provincial transfer of grain carbon emissions and its reduction efficiency of carbon emissions, and pointed out that the provincial supply of grain has more reduction efficiency of carbon emissions [39].

The previous research results of the academic circle on carbon emissions and carbon transfer provide important ideas and methodical references for further in-depth research on carbon transfer and its spatial pattern of China’s inter-provincial agricultural trade, but it still needs to be further strengthened in the following aspects:

1. Existing studies on agricultural trade pay more attention to virtual land and water resource transfer yet insufficient attention to the implied carbon emissions transfer attached to its process. Moreover, studies on agricultural implied carbon transfer pay more attention to food trade, while pay less attention to the carbon transfer caused by animal product trade, which cannot reflect the overall picture of agricultural carbon transfer.
2. In terms of research methods, due to the difficulty in obtaining inter-regional agricultural trade data [40], the multi-regional input-output model is generally adopted for calculation [41]. However, in this paper’s study of inter-provincial agricultural carbon transfer, the multi-regional input-output method exhibits certain limitations. Firstly, China lacks an accurate statistical system for compiling multi-regional input-output (MRIO) tables. To construct an MRIO table, it is necessary to initially estimate the regional trade matrix using a gravity model, meaning that the MRIO model also relies on estimation methods. Secondly, the input-output table is updated every five years, which results in relatively poor timeliness when used for research and will affect the time efficiency and limitation of the input-output model. For instance, Ren et al. (2018) conducted their study using multi-regional input-output data from 2007, which was 11 years behind the year of their research [42]. Lastly, China’s published regional input-output tables do not provide a detailed breakdown for agriculture, and there are significant differences in the agricultural production structure among different regions, with some focusing primarily on grain cultivation and others on livestock and poultry farming. Additionally, there are considerable differences in the consumption structure of agricultural products across regions. Consequently, while the multi-regional input-output model can reflect inter-provincial trade connections in regional agriculture, it struggles to represent more specific trade information for agricultural products, posing difficulties in estimating agricultural carbon transfer using this model.

Given this, the paper will adopt a linear programming model. The linear programming model quantifies agricultural trade from the perspective of driving factors, and eliminates the limitations of agricultural trade data, and provides a new and feasible reference for the study of carbon emission transfer in agricultural trade process, which has been confirmed by many scholars at home and abroad [43].

On one hand, the linear programming model can categorize agriculture into cultivation and animal husbandry, allowing for the separate estimation of carbon transfer in the trade of grain and meat agricultural products. On the other hand, agricultural product trade not only achieves a balance of supply and demand across different regions but also inherently seeks to minimize transportation costs during the trade process. Consequently, the transportation cost of agricultural products is a primary driver affecting trade. As the linear programming model has been widely applied in the field of agricultural trade, numerous studies have expanded on the driving factors of agricultural trade, including trade costs, transportation costs, and opportunity costs [44–46]. These studies play a significant role in broadening the application of the linear programming model in agricultural trade. However, traditional linear programming models for agricultural products often presume uniform production costs across regions, overlooking the variability in regional production costs. In this research, the linear programming model has been refined to incorporate regional production costs of agricultural products, recognizing that trade is influenced not only by transportation costs related to distance but also by the differences in production costs among regions. The linear programming model can differentiate between grain and meat, estimating agricultural carbon transfer through inter-provincial trade calculations. Compared to the multi-regional input-output model, the linear programming model is a "bottom-up" approach that offers a more precise estimation of agricultural carbon transfer issues.

In view of this, the study first used agricultural carbon metering models to calculate agricultural carbon emissions in 2015, 2018 and 2021 in 31 provincial-level administrative regions in China (excluding Hong Kong, Macao and Taiwan). Then, a linear programming model was constructed to simulate the inter-provincial trade of food and animal products based on the comprehensive consideration of the production cost and transportation cost of inter-provincial agricultural products, and the overall carbon emission transfer of agriculture was comprehensively identified. On this basis, it analyzed the temporal and spatial characteristics of carbon transfer in agricultural trade at provincial scale for the purpose of providing a basis for formulating fair reduction policies of agricultural inter-provincial carbon emissions.

## Method and materials

Accurate measurement of agricultural carbon emissions is the foundation of inter-provincial agricultural carbon transfer. It is necessary to clarify accurate measurement methods of agricultural carbon emissions in different provinces in the first place and to estimate carbon emissions in different provinces according to existing statistical data. The study inter-provincial trade of agricultural products shall be conducted on the base of inter-provincial carbon emissions accounting, then the amount and direction of carbon transfer attached to agricultural products can be estimated.

### Carbon accounting model

According to the IPCC guidelines and related research results, this paper constructs carbon emissions accounting accounts from the perspective of planting industry and livestock and poultry industry. It applies coefficient measurement method to estimate agricultural carbon emissions. The agricultural carbon accounting model is as follows:

$$C\;=\;\sum C_{i}\;=\;\sum T_{i}∙\mu_{i}$$ (1)

Among which, *C* is the total carbon emissions of agriculture from planting industry and livestock and poultry industry. *C*_*i*_ means the emission volume of Class *i* carbon source. *T*_*i*_ represents the number of Class *i* carbon source with *μ*_*i*_ as its emission coefficient. When the IPCC calculation principles are applied to agriculture, greenhouse gases are the agricultural activities that produce greenhouse gases multiplied by the corresponding emission factor.

Specifically, greenhouse gas emissions from crop cultivation mainly include CH_4_ emissions from rice, N_2_O emissions from crop growth and carbon emissions from input of agricultural materials. The measured crop varieties include rice (early rice, middle rice, late rice), wheat (spring wheat, winter wheat), soybean, corn, vegetables, oil, sugar and other dryland crops. The types of agricultural input mainly include chemical fertilizer, pesticide, agricultural film, diesel, agricultural irrigated area, ploughing and so on. The greenhouse gas emissions from livestock breeding mainly include the emissions from fermentation in the gastrointestinal tract of livestock and from livestock and poultry manure. Measured breeds include cows, buffaloes, cattle, horses, donkeys, mules, camels, pigs, sheep, rabbits, poultry and so on. Further, this paper converts various greenhouse gases to carbon dioxide equivalents according to international standards.

### Carbon transfer model

Inter-provincial trade of agricultural products is affected by supply and demand, price, transportation price, transportation distance and regional consumption structure. Therefore, aiming at minimum trade cost as the objective function, a linear programming model is built to simulate the flow of inter-provincial agricultural trade in this paper. This paper does not consider the international trade of agricultural products for the time being, so it excludes the import and export trade of agricultural products. At the same time, it is assumed that there is no difference in the quality of similar agricultural products produced by different provinces, and provinces only transfer rich agricultural products to those in shortage. The specific calculation formula is as follows:

$$Min(\sum_{i\;=\;1}^{N}\sum_{j\;=\;1}^{M}x_{ij}∙(F_{j}^{c}+F_{ij}^{t}))$$ (2)

$$F_{ij}^{t}\;=\;F_{1}^{k}+F_{2}^{k}∙d_{ij}$$ (3)

$$\sum_{i\;=\;1}^{N}x_{ij}\leq S_{j}$$ (4)

$$\sum_{j\;=\;1}^{M}x_{ij}\;=\;D_{i}$$ (5)

$$x_{ij}\geq 0$$ (6)

where, equation (2) indicates the minimized transportation costs for inter-provincial agricultural trade; *x*_*ij*_ is the number of agricultural products transported from province *j* to province *i*, *N* means the total number of provinces with shortage of agricultural products, *M* is the total number of remaining provinces of agricultural products, *N*+*M* = 31; $\;F_{j}^{c}$ is the agricultural production cost of province *j*, $\;F_{ij}^{t}$ is the transportation cost of agricultural products transported from province *j* to province *i*, *d*_*ij*_ is the transportation distance of province *j* to province *i*, $\;F_{1}^{k}$ is the basic transportation cost for *k* agricultural products, and $F_{2}^{k}$ is unit transportation cost per kilometer for K agricultural products; *D*_*i*_ and *S*_*j*_ mean the shortage of agricultural products in province *i* and the surplus of agricultural products in province *j*.

According to the linear programming model of agricultural trade, it can calculate the optimal number *X_xj_* of agricultural products transported from province *j* to provinces *i*, thus it can further calculate the carbon transfer caused by the inter-provincial circulation of agricultural products as follows:

$$C_{ij}\;=\;x_{ij}∙\varphi_{j}$$ (7)

$$C_{j}\;=\;\sum_{i}C_{ij}$$ (8)

*C*_*ij*_ is the carbon emissions of the agricultural product *x*_*ij*_ produced by province *j* and consumed by province *i*. *φ*_*j*_ is carbon emission coefficient of the *x*_*ij*_ produced by province *j*, and *C*_*j*_ means the sum of carbon emission transported to other provinces from *j* province.

### Data and description

The data of crop sown area, output, input of agricultural materials, stock of livestock and poultry breeding and the quantity of livestock and poultry were derived from China Agricultural Yearbook, China Rural Statistical Yearbook, China Statistical Yearbook, China Animal Husbandry and Veterinary Yearbook, etc. GHG emission coefficient of crops, GHG emission coefficient of input of agricultural materials, GHG emission coefficient of animal gastrointestinal fermentation and GHG emission coefficient of animal husbandry are mainly obtained by referring to previous studies [47–61].

The carbon emissions of planting industry are mainly from food carbon emissions, and the carbon emissions of livestock and poultry farming are mainly from cattle, pigs, and sheep. The calculations results indicate that that during the study period, the carbon emissions of grain accounted for about 80% of the carbon emissions of planting industry, and the carbon emissions of cattle, sheep and pig farming accounted for about 90% of the carbon emissions of all livestock and poultry farming. This paper only calculates the circulation of grain (grains, beans, potatoes) and meat (pork, beef, lamb) products, which is used to delegate the transfer of carbon emissions from planting and livestock and poultry farming. Food consumption includes five categories: ration consumption, feed consumption, industrial consumption, seed grain and grain loss. Ration consumption includes household food consumption and non-household food consumption. Household food consumption is calculated by multiplying the per capita food consumption by the resident population of each region. For urban residents, the proportion of non-household food consumption is 4% while the number becomes 12% for rural residents. Feed consumption is converted according to the output of livestock and poultry products in each province according to the feed conversion rate. Industrial consumption is estimated according to a certain conversion ratio, and seed consumption is converted according to the sown area of grain varieties in each province. The conversion rate and per-unit seed consumption are shown in Table 1 [62]. Grain loss includes inventory loss, transportation loss and processing loss. Inventory loss is calculated according to 2% of grain production. Transportation loss and processing loss are calculated according to 4‰ and 5‰ of the sum of ration, forage and industrial consumption in grain consumption respectively [63]. Meat consumption includes meat consumption in urban and rural households and meat consumption outside urban and rural households. And with the acceleration of economic development, the proportion of meat consumption outside the household has increased rapidly. The proportion of meat consumption outside the household of urban residents and the proportion of meat consumption outside the household of rural residents in China were obtained with reference to previous studies [64]. Considering that there is a certain loss of meat products in transportation, storage, processing and other links, the total loss rate of meat products is 6.4% of its total output [65]. The meat production data is carcass weight, while the consumption data are net weight, there are certain differences, and this study classifies them as other consumption. In addition, it is assumed that the agricultural surpluses of all regions are transferred to other regions, and all agricultural deficits are made up by imports [46, 66].

**Table 1:** Conversion factor of food consumption.

Feed	Pork	Beef & mutton	Poultry	Eggs	Fish
	1:3.5	1:1.7	1:1.7	1:2.2	1:0.9
Industrial	Alcohol	White wine	Beer	Monosodium glutamate	
	1:3	1:2.3	1:0.172	1:2.4	
Seed (kg/ha)	Rice	Corn	Soybean	Potato	The rest
	75	75	750	120	225

The transportation price of agricultural goods shall refer to the Benchmark Freight Rate Table of Various Goods Railway Transportation issued by the National Development and Reform Commission in 2015. The production cost data of grain and meat products were derived from the Compilation of National Agricultural Product Cost-Benefit Information, and the missing data were replaced by the national average. The transportation distance of agricultural products comes from the "Railway Passenger Fare Odometer" published by China National Railway Group Co., LTD. The transportation distance is based on the odometer of major railway stations in the country, and some provinces are supplemented by the minimum mileage of provincial capital city stations in the national mileage query of railway stations.

Due to data limitations, this paper did not compile product balance tables for other crops such as vegetables, tea, oil, and other animal products such as donkeys, camels, and rabbits. Therefore, when calculating the inter-provincial trade of agricultural products, it is assumed that the inter-provincial trade of non-food crops is consistent with the trade of grain, and the trade of other meat is consistent with the trade of meat (beef, mutton, pork). Based on this, it further estimates the overall situation of agricultural carbon transfer.

## Results

### Analysis of interprovincial agricultural carbon transfer in China

**Fig 1:**
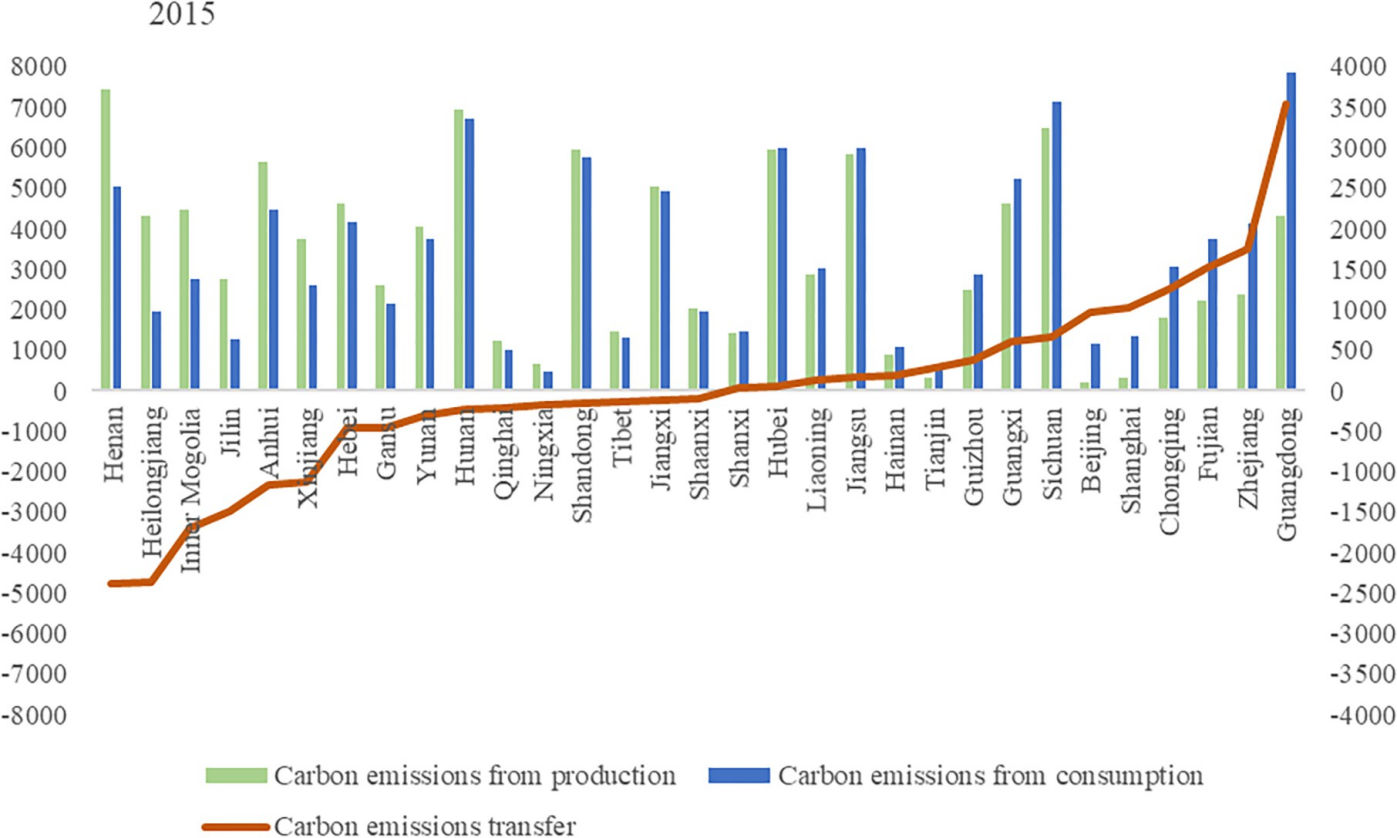
Interprovincial agricultural carbon emission and carbon transfer in 2015. (**Unit:** ten thousand tons of CE).

**Fig 2:**
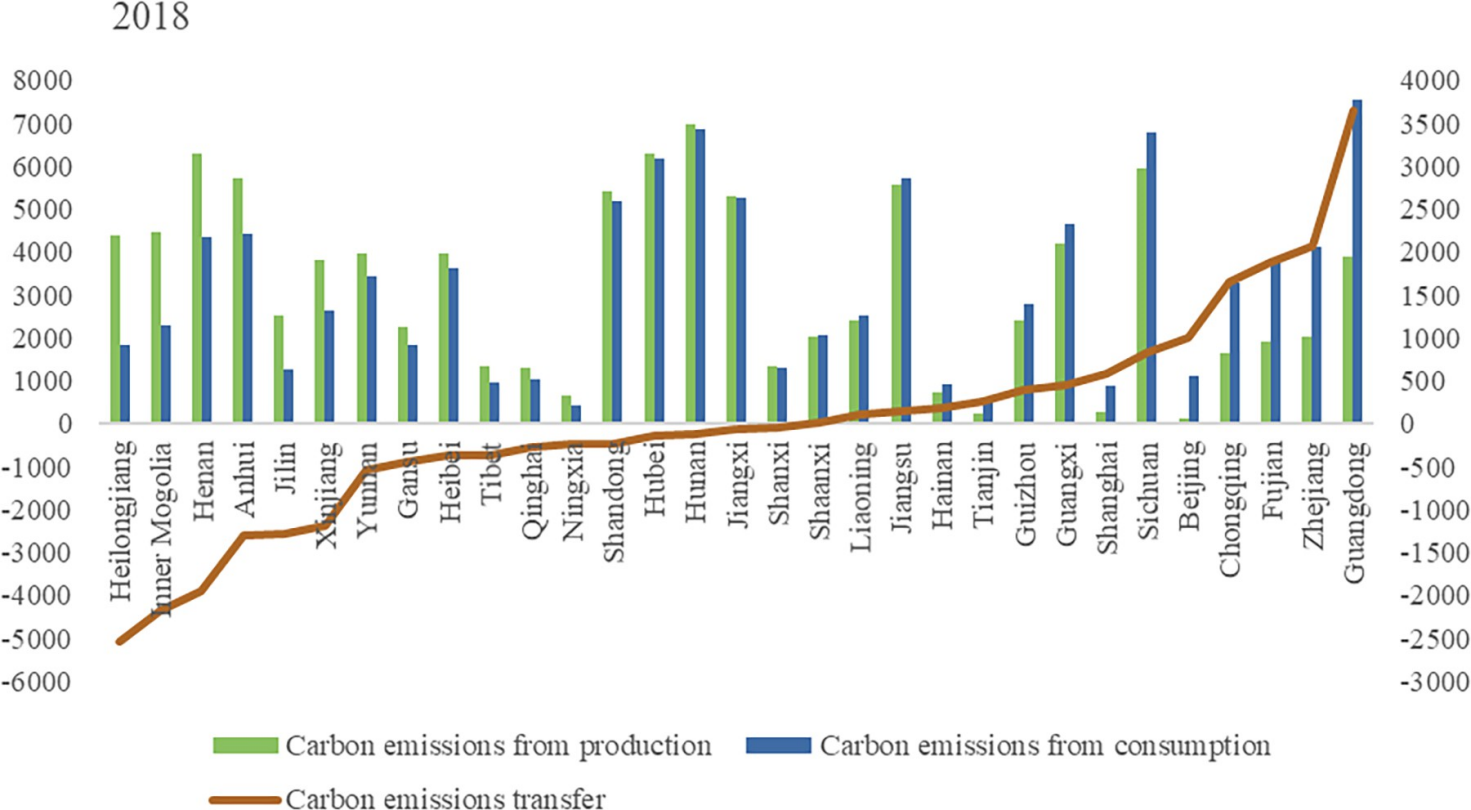
Interprovincial agricultural carbon emission and carbon transfer in 2018. (**Unit:** ten thousand tons of CE**).**

**Fig 3:**
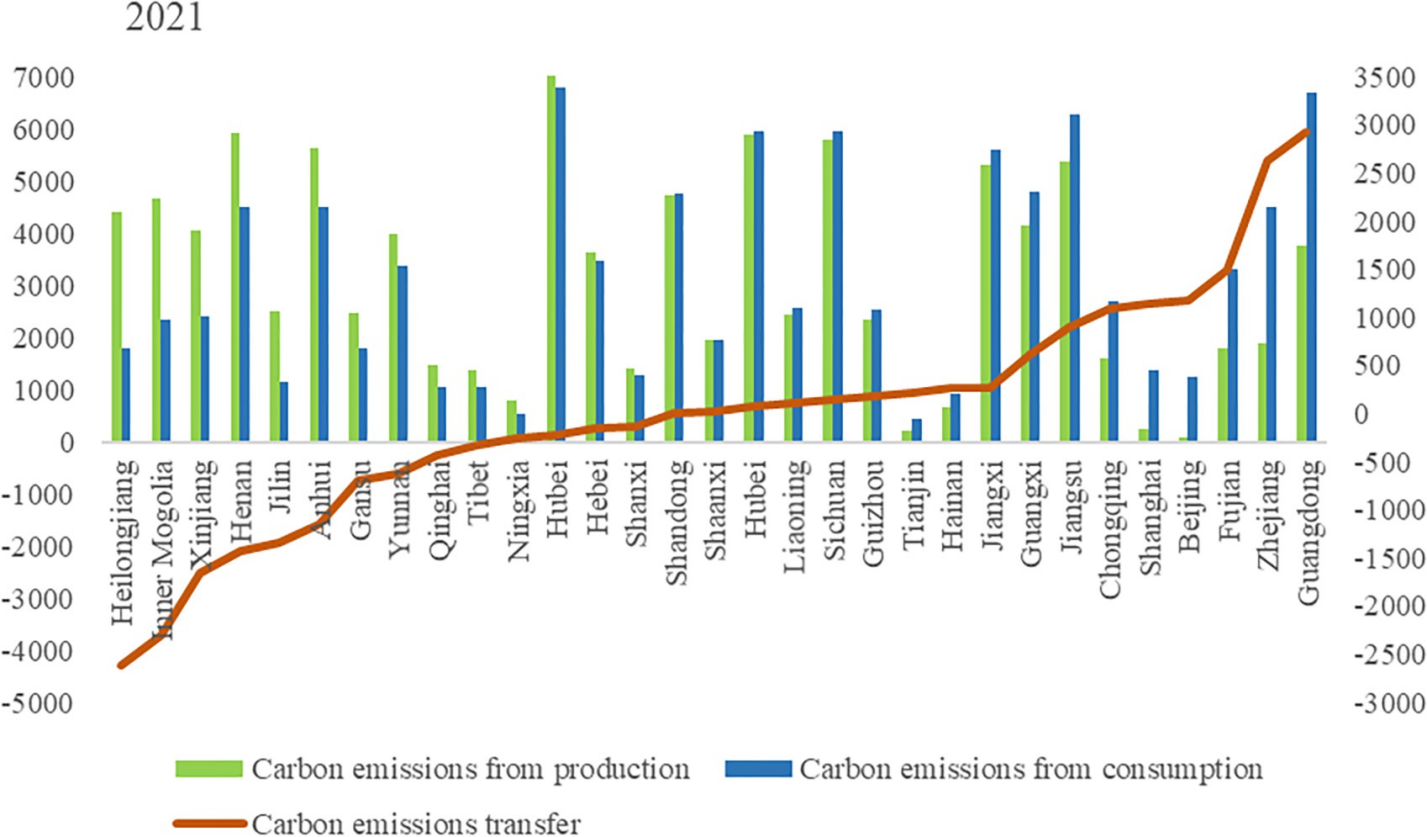
Interprovincial agricultural carbon emission and carbon transfer in 2021. (**Unit:** ten thousand tons of CE**).**

Carbon emissions from agricultural production mainly come from crop farming, and livestock and poultry farming. Based on the carbon emissions accounting method, this paper calculates the carbon emissions of production and consumption in various provinces in China. Figs 1–3 shows the inter-provincial agricultural carbon emissions and carbon transfer in both sides of production and consumption in 2015, 2018 and 2021.

Overall, the top 13 provinces with agricultural carbon emissions from production side accounted for 68.17%, 68.89% and 68.61% of the total agricultural carbon emissions of the country in 2015, 2018 and 2021 respectively. China’s 13 major grain producing provinces are Heilongjiang, Henan, Shandong, Sichuan, Jiangsu, Hebei, Jilin, Anhui, Hunan, Hubei, Inner Mongolia, Jiangxi and Liaoning. Agricultural carbon emissions from these provinces accounted for 65.03%, 65.66% and 64.86% of the country’s total agricultural carbon emissions in 2015, 2018 and 2021 respectively. We found that the provinces with high agricultural carbon emissions are highly overlapping with major grain-producing regions in China. The superior agricultural production conditions and relatively developed planting and breeding industries in major grain-producing regions are the main reasons for the high level of agricultural carbon emissions. It is worth noting that although Xinjiang, Yunnan, Gansu and other regions are not major grain-producing regions, their agricultural carbon emissions have exceeded some major grain-producing provinces. To illustrate the reasons, this article provides data on carbon emissions from grain and livestock farming in Table 2. The main reason is that the livestock and poultry breeding industry in areas such as Xinjiang, Yunnan, and Gansu is more developed, leading to higher carbon emissions from livestock and poultry breeding. In particular, Tibet and Qinghai have livestock and poultry breeding carbon emissions that far exceed their grain production carbon emissions.

**Table 2:** Agricultural carbon emission volumes of different provinces in 2021 (Unit: Million tons of CE).

	Planting Industries	Livestock and Poultry Breeding Industries	Total Carbon Emissions
Beijing	0.54	0.26	0.80
Tianjin	1.20	1.02	2.22
Hebei	20.84	15.42	36.26
Shanxi	7.80	6.41	14.21
Inner Mongolia	17.62	28.98	46.60
Liaoning	12.85	11.64	24.49
Jilin	15.61	9.29	24.90
Heilongjiang	29.62	14.53	44.15
Shanghai	2.10	0.31	2.41
Jiangsu	48.56	5.13	53.69
Zhejiang	16.97	1.92	18.89
Anhui	48.60	7.86	56.46
Fujian	13.98	4.01	17.99
Jiangxi	43.94	9.32	53.26
Shandong	30.34	17.04	47.38
Henan	39.42	19.99	59.41
Hubei	47.85	10.99	58.84
Hunan	52.52	17.78	70.30
Guangdong	29.80	7.91	37.71
Guangxi	29.68	11.93	41.61
Hainan	4.91	1.65	6.56
Chongqing	10.86	5.26	16.12
Sichuan	28.67	29.32	57.99
Guizhou	11.19	12.28	23.47
Yunnan	15.74	24.31	40.05
Tibet	0.40	13.28	13.68
Shaanxi	13.03	6.40	19.43
Gansu	9.51	15.26	24.77
Qinghai	0.72	14.03	14.75
Ningxia	2.84	5.26	8.10
Xinjiang	17.76	22.83	40.59

The low level of agricultural carbon emissions in Beijing, Tianjin, Shanghai, Hainan and Ningxia is mainly due to the constrains of agricultural production conditions in these regions. Regions with small area like Beijing, Tianjin and Shanghai are not suitable for large-scale agricultural development. Hainan mainly develops tropical agriculture due to the limitation of climatic conditions. While the both of the planting and breeding industries are underdeveloped in Ningxia because of its poor agricultural production conditions such as the small cultivated land area and the water shortage.

The top 13 provinces with agricultural carbon emissions on the consumption side accounted for 71.57%, 68.79%and 68.80% of the total agricultural carbon emissions of the country in 2015, 2018 and 2021 respectively. Provinces with higher agricultural carbon emissions on the consumption side are Guangdong, Sichuan, Hunan, Hubei, Jiangxi, Jiangsu, Shandong, Henan, Hebei, Zhejiang, Anhui, Fujian and Yunnan. These provinces are mainly distributed in the Yangtze River Economic Belt and the eastern coastal areas, which are economically developed areas with large population and resulted in more agricultural carbon emissions on the consumption side.

Figs 1–3 also show the amount of carbon transfer caused by agricultural trade. Negative carbon transfer indicates that the province belongs to the net output area of agricultural carbon emissions, which means that the agricultural carbon emissions on the consumption side is less than that on the production side. Positive carbon transfer indicates that the province is a net input area of agricultural carbon emissions, which means that the agricultural carbon emissions on the consumption side are greater than that on the production side. From the perspective of total agricultural carbon transfer, in 2015, 2018 and 2021, agricultural carbon transfer accounted for 11.97%, 13.31% and 13.61% of total agricultural carbon emissions respectively, and the proportion increased slightly. From the perspective of inter-provincial agricultural carbon transfer, Heilongjiang, Inner Mongolia, Xinjiang, Henan, Anhui, Jilin, Gansu, Hebei, Yunnan, Tibet, Ningxia, Qinghai and Hunan were net agricultural carbon emissions exporting regions in 2015, 2018 and 2021, while Guangdong, Zhejiang, Fujian, Chongqing, Sichuan, Beijing, Shanghai, Guangxi, Guizhou, Tianjin, Hainan, Jiangsu, and Liaoning were net contributors to agricultural carbon emissions in 2015, 2018 and 2021.

### Characteristics of interprovincial agricultural carbon transfer flow

Table 3 shows the cumulative share of agricultural carbon emissions input area and output area according to the total ranking, and there are obvious agglomeration characteristics in both the net input area and the net output area of agricultural carbon emission. Specifically, the top 5 provinces accounted for 72.28%, 77.58% and 70.52% of the total net agricultural carbon input in 2015, 2018 and 2021 respectively. The top 5 provinces and regions accounted for 72.37%, 69.78% and 70.04% of the total net agricultural carbon output, respectively.

**Table 3:** Proportion of agricultural carbon transfer in different provinces (Unit: %).

2015			2018			2021		
Net Input	Per(%)	Cum(%)	Net Input	Per(%)	Cum(%)	Net Input	Per(%)	Cum(%)
Guangdong	28.09	28.09	Guangdong	27.58	27.58	Guangdong	22.06	22.06
Zhejiang	13.93	45.01	Zhejiang	15.68	43.26	Zhejiang	19.73	41.80
Fujian	12.17	54.18	Fujian	14.26	57.52	Fujian	11.26	53.06
Chongqing	9.99	64.18	Chongqing	12.52	70.04	Beijing	8.83	61.89
Shanghai	8.10	72.28	Beijing	7.55	77.58	Shanghai	8.63	70.52
Beijing	7.74	80.02	Sichuan	6.25	83.83	Chongqing	8.15	78.67
Sichuan	5.25	85.26	Shanghai	4.39	88.22	Jiangsu	6.76	85.43
Guangxi	4.78	90.05	Guangxi	3.36	91.59	Guangxi	4.70	90.13
Guizhou	3.03	93.08	Guizhou	3.03	96.62	Jiangxi	2.04	92.18
Tianjin	2.22	95.30	Tianjin	1.93	96.55	Hainan	1.97	94.15
Hainan	1.51	96.81	Hainan	1.35	97.89	Tianjin	1.64	95.78
Jiangsu	1.31	98.12	Jiangsu	1.17	99.06	Guizhou	1.42	97.21
Liaoning	1.08	99.19	Liaoning	0.84	99.91	Sichuan	1.09	98.30
Hubei	0.44	99.63	Shaanxi	0.09	100.00	Liaoning	0.88	99.18
Shanxi	0.37	100.00				Hubei	0.61	99.79
						Shaanxi	0.13	99.92
						Shandong	0.08	100.00
Net Output	Per(%)	Cum(%)	Net Output	Per(%)	Cum(%)	Net Output	Per(%)	Cum(%)
Henan	18.96	18.96	Heilongjiang	19.17	19.17	Heilongjiang	19.65	19.65
Heilongjiang	18.77	37.73	Inner Mongolia	16.40	35.57	Inner Mongolia	17.33	36.97
Inner Mongolia	13.54	51.27	Henan	14.72	50.29	Xinjiang	12.37	49.34
Jilin	11.81	63.08	Anhui	9.79	60.08	Henan	10.69	60.04
Anhui	9.30	72.37	Jilin	9.71	69.78	Jilin	10.00	70.04
Xinjiang	8.95	81.32	Xinjiang	8.96	78.74	Anhui	8.61	78.65
Hebei	3.67	85.00	Yunnan	4.13	82.87	Gansu	5.20	83.86
Gansu	3.67	88.66	Gansu	3.35	86.22	Yunnan	4.64	88.50
Yunnan	2.47	91.14	Hebei	2.84	89.06	Qinghai	3.29	91.79
Hunan	1.83	92.97	Tibet	2.75	91.81	Tibet	2.47	94.26
Qinghai	1.61	94.58	Qinghai	2.07	93.87	Ningxia	1.98	96.24
Ningxia	1.44	96.02	Ningxia	1.77	95.64	Hunan	1.66	97.90
Shandong	1.23	97.25	Shandong	1.73	97.36	Hebei	1.12	99.03
Tibet	1.15	98.40	Hubei	0.98	98.35	Shanxi	0.97	100.00
Shaanxi	0.74	99.13	Hunan	0.97	99.32			
Jiangxi	0.87	100.00	Hubei	0.55	99.12			
			Jiangxi	0.41	99.74			
			Shanxi	0.26	100.00			

On the whole, the agricultural carbon transfer is spatially divided by the Yangtze River, and the areas north of the Yangtze River are mostly net agricultural carbon emissions output areas, while the areas south of the Yangtze River are mostly net agricultural carbon emissions input areas. The carbon emissions flow pattern of agricultural trade is highly correlated with the mismatch between agricultural production capacity and consumption capacity in China. At present, as the economic center of gravity is mainly distributed in the Beijing-Tianjin-Hebei, Yangtze River Delta, Pearl River Delta and Chengdu-Chongqing region. The highly developed industry and services indicate their large demand for agricultural products in these regions. From the perspective of agricultural production, Heilongjiang, Henan, Anhui, Inner Mongolia and other regions have superior agricultural production conditions and high output of agricultural products. The uneven spatial distribution of the demand and supply of agricultural products is the root cause of the inter-provincial agricultural trade, and it also provides conditions for the allocation and optimization of natural resources by the market.

**Fig 4:**
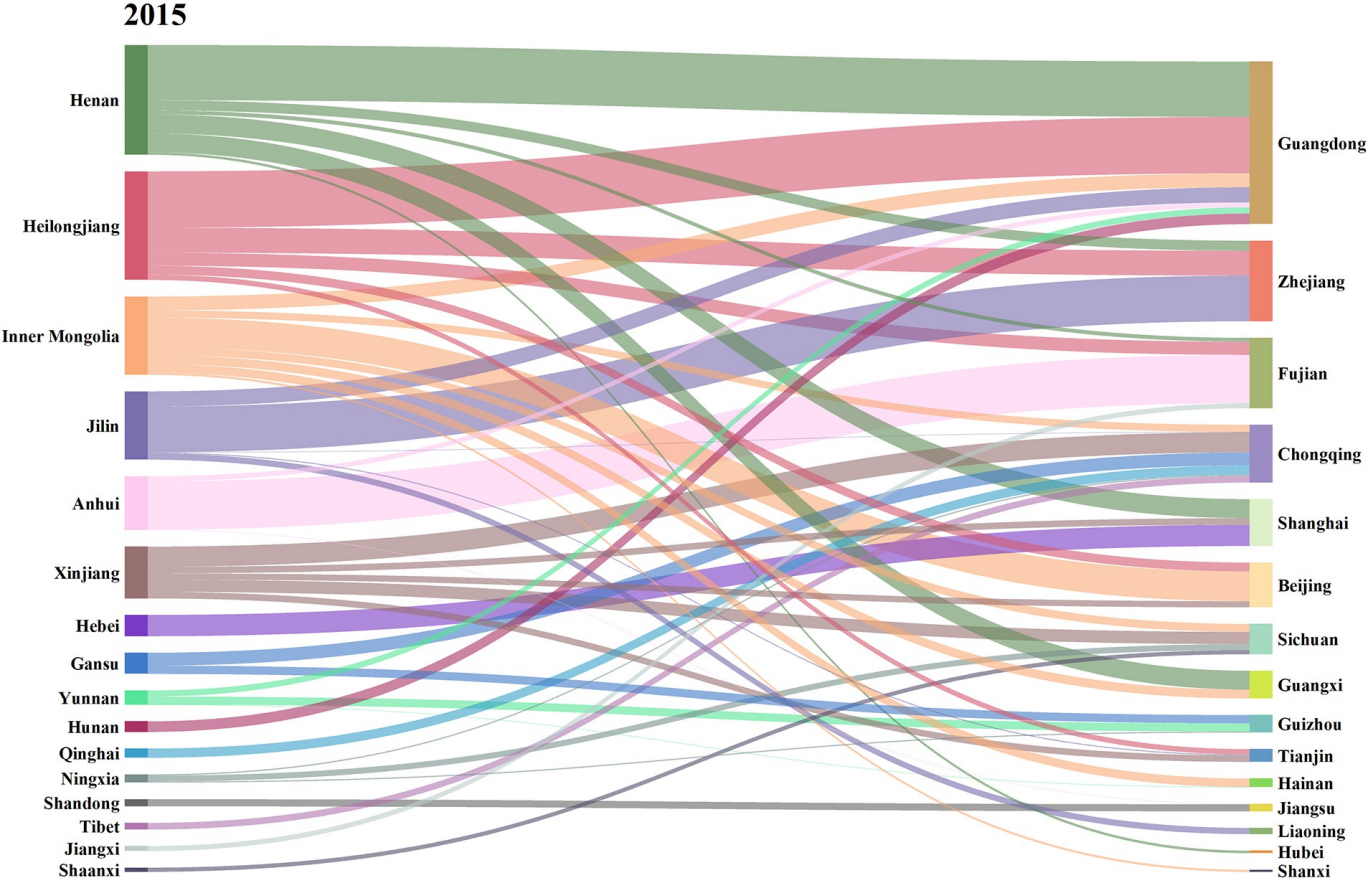
Carbon emissions flows in agricultural trade in 2015. (**Unit:** ten thousand tons of CE**).**

**Fig 5:**
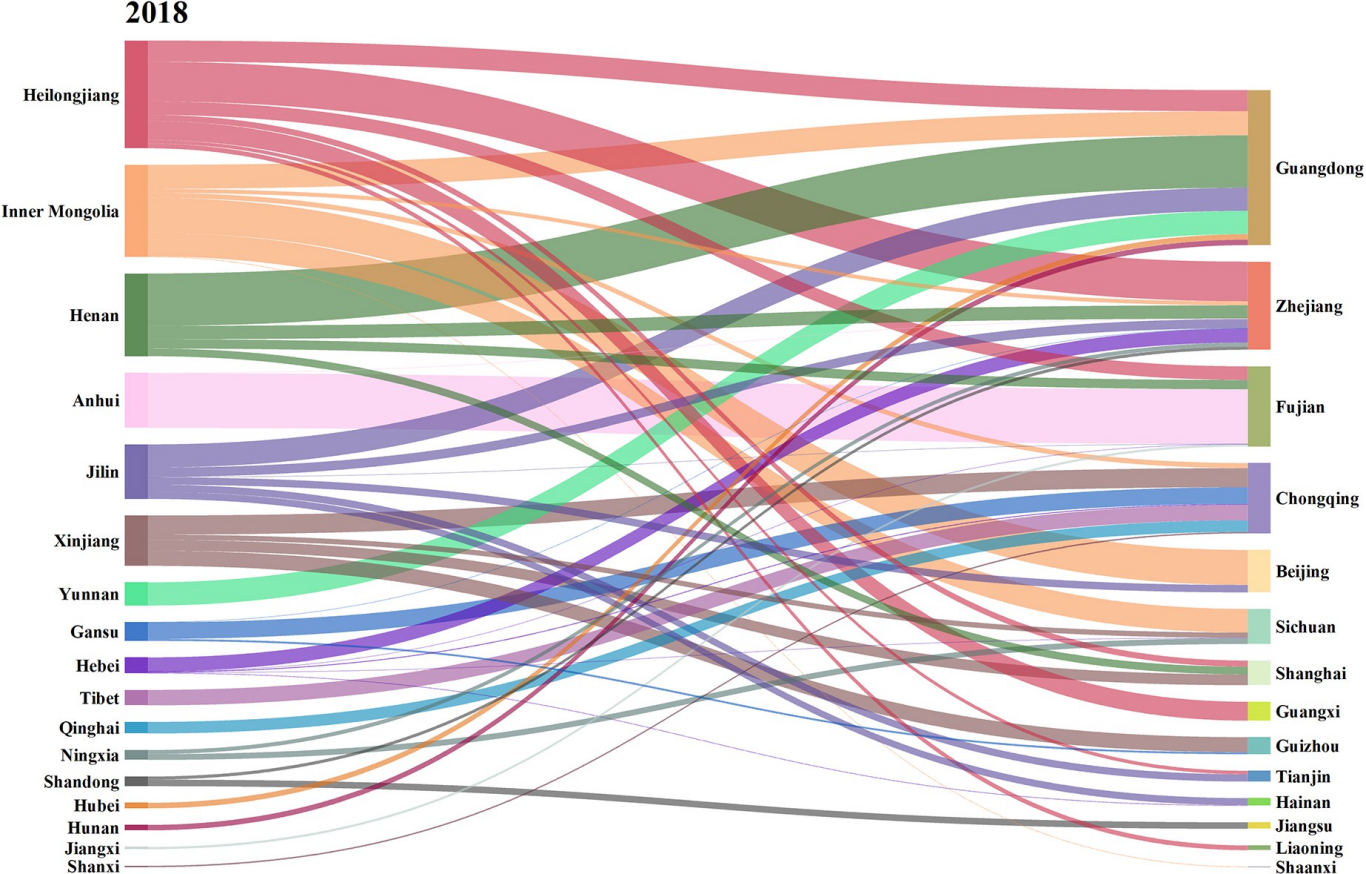
Carbon emissions flows in agricultural trade in 2018. (**Unit:** ten thousand tons of CE**).**

**Fig 6:**
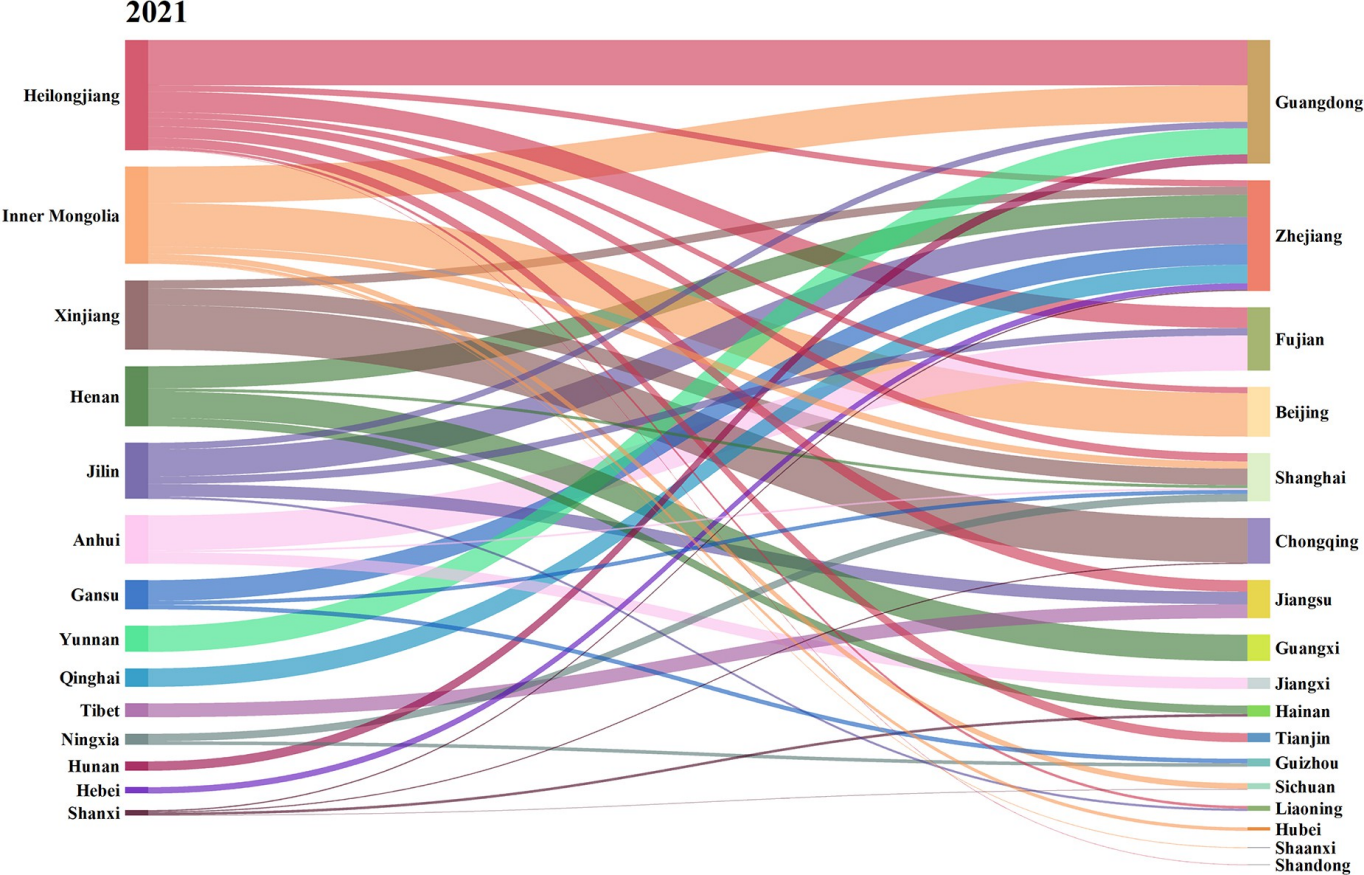
Carbon emissions flows in agricultural trade in 2021. (**Unit:** ten thousand tons of CE**).**

Further, this paper calculates the net carbon input and output of agricultural trade in 31 provinces in China in 2015, 2018 and 2021, as shown in Figs 4–6. The left side represents the net output province of agricultural carbon emissions, and the right side represents the net input province.

From the changes of inter-provincial agricultural carbon transfer in different years, the flow direction and flow characteristics of agricultural carbon emissions showed high similarity despite differences in 2015 and 2018. Compared with 2015 and 2018, the characteristics of agricultural carbon emissions flow have undergone great changes in 2021. Guangdong and Zhejiang, the two provinces with the largest net import, were taken as examples. In 2015 and 2018, the agricultural carbon emissions imported from Guangdong mainly came from Henan, Inner Mongolia, Jilin and Heilongjiang. In 2015, the agricultural carbon emissions exported from Henan, Heilongjiang, Jilin and Inner Mongolia to Guangdong accounted for 34.01%, 34.72%, 9.36% and 8.55% of the total agricultural carbon emissions imported from Guangdong, respectively, while the proportion were 33.78%, 13.67%, 14.96% and 15.51% in 2018. In 2021, the agricultural carbon emissions imported into Guangdong mainly came from, Heilongjiang, Inner Mongolia and Yunnan, accounting for 36.63%, 29.47%, 21.02% of the total agricultural carbon emissions imported into Guangdong respectively. This phenomenon may be caused by the following two reasons. Firstly, in 2015 and 2018, Henan’s agricultural carbon transfer to Guangdong was mainly through the trade of meat products from the livestock and poultry breeding industry. The net exports of meat products from Henan were 2.25 million tons and 1.86 million tons in 2015 and 2018, respectively, but by 2021, the net export of meat products from Henan decreased to 1.00 million tons. Due to the significant decrease in the net export of meat products, Henan’s supply capacity for meat products to Guangdong substantially decreased, leading to a reduction in the amount of agricultural carbon transfer. Secondly, as Henan’s meat industry declined, Guangdong began to transfer in meat products from Yunnan.

In 2015 and 2018, the agricultural carbon emissions imported into Zhejiang mainly came from Heilongjiang, Jilin and Henan, accounting for over 70.00% of the total agricultural carbon emissions. In 2021, the agricultural carbon emissions exported to Zhejiang mainly came from Heilongjiang, Henan and Jilin, accounting for 50.19% of the total agricultural carbon emissions imported from Zhejiang. In 2021, the main provinces contributing to the agricultural carbon emissions entering Zhejiang included Gansu and Qinghai, which were an addition compared to 2015 and 2018. The main reason is the increasing demand gap for meat products in Zhejiang. In addition to importing meat products from Henan, Heilongjiang, Jilin, it is also necessary to import from regions such as Qinghai and Gansu.

Henan and Inner Mongolia are typical agricultural carbon emissions exporting provinces. In 2015 and 2018, Henan’s agricultural carbon emissions mainly flowed into Guangdong, accounting for 50.37% and 63.28% of Henan’s total agricultural carbon emissions, respectively. In 2021, the agricultural carbon emissions exported from Henan mainly flowed into Guangxi Zhejiang and Hainan, accounting for 43.99%, 36.92% and 14.10% of the total agricultural carbon emissions, respectively. The proportion of agricultural carbon emissions exported from Henan to Guangdong decreased significantly, and the output destination became average and diversified. After 2021, there was a significant decline in Henan’s meat supply capacity, and the overall carbon emissions from agricultural trade in Henan decreased, but its agricultural carbon emission export destination is still neighboring provinces in Guangdong.

In 2015 and 2018, the agricultural carbon emissions of Inner Mongolia mainly flowed to Guangdong, Beijing. In 2015, the agricultural carbon emissions of Inner Mongolia to Guangdong and Beijing accounted for 17.74% and 37.96% of its total output, respectively. In 2018, the agricultural carbon emissions of Inner Mongolia to Guangdong and Beijing accounted for 26.08% and 37.98% of its total output, respectively, while in 2021, Inner Mongolia still maintained this pattern. In 2021, the agricultural carbon emissions of Inner Mongolia to Guangdong and Beijing accounted for 37.52% and 44.81% of its total output, respectively.

### Analysis of characteristics of interprovincial agricultural carbon transfer path

According to the of agricultural carbon transfer and spatial distribution of the major provinces (cities), this paper divides the net input and output regions of agricultural carbon emissions into four major regions, as shown in Table 4.

**Table 4:** Regional division of agricultural carbon transfer.

Input Regions for Carbon Emissions	I-Ⅰ	Shanghai, Zhejiang, Fujian
	I-Ⅱ	Guangdong, Guangxi
	I-Ⅲ	Chongqing, Sichuan, Guizhou
	I-Ⅳ	Beijing, Tianjin
Output Regions for Carbon Emissions	O-Ⅰ	Henan, Anhui
	O-Ⅱ	Heilongjiang, Jilin
	O-Ⅲ	Inner Mongolia
	O-Ⅳ	Xinjiang, Gansu

The "I-Ⅰ" area refers to the eastern coastal area with Zhejiang as the core, including Zhejiang, Shanghai, and Fujian. The "I-II" area refers to the southern coastal area with Guangdong as the core, including Guangdong and Guangxi. The "I-III" region indicates the upper reaches of the Yangtze River with Chongqing as the core, including Chongqing, Sichuan and Guizhou. The "I-IV" area covers the Beijing-Tianjin area with Beijing as the core, including Beijing and Tianjin. China’s net agricultural carbon output area can also be roughly divided into four regions. The "O-Ⅰ" region includes Henan, Anhui provinces with Henan as the core. The "O-II" region contains Heilongjiang and Jilin provinces with Heilongjiang as the core. The "O-Ⅲ" region is Inner Mongolia. The "O-Ⅳ" region means Xinjiang and Gansu with Xinjiang as the core.

**Fig 7:**
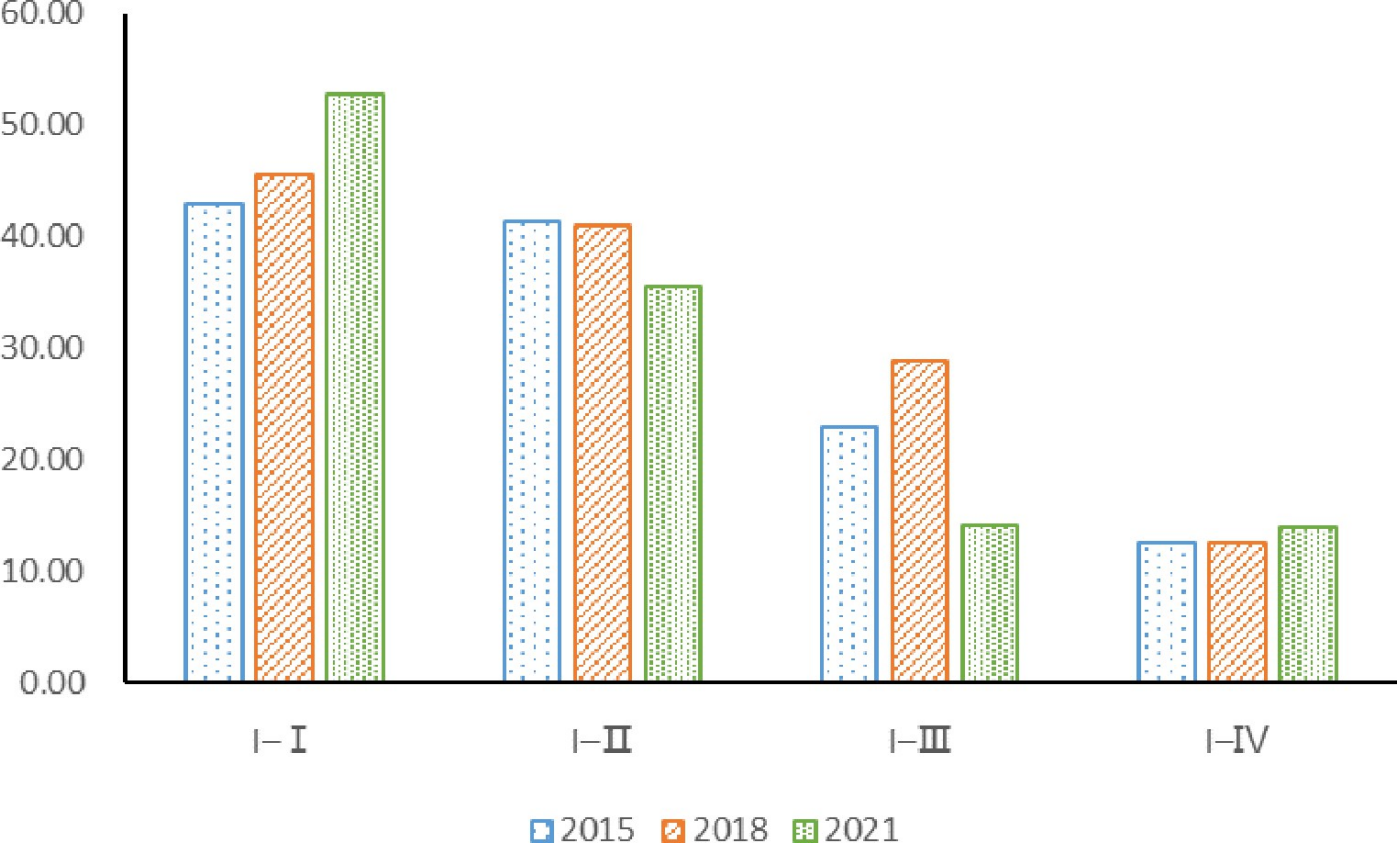
Carbon emissions from agricultural trade in major net importing regions. (**Unit:** million tons of CE**).**

Fig 7 shows regional agricultural carbon emissions input, "I-I" is the region with the largest net carbon emissions input from China’s agricultural trade, followed by "I-II" and "I-IV". Therefore, the net input area of agricultural carbon emissions in China is mainly concentrated in the south of the Yangtze River spatially. In 2021, the net carbon emissions input of "I-I", "I-II", "I-III" and "I-IV" were 52.71 million tons, 35.61 million tons, 14.20 million tons and 13.92 million tons, accounting for 39.62%, 26.77%, 10.67% and 10.46% of the total net carbon emissions input respectively. Comparing with 2015 and 2018, the net input of agricultural carbon emissions from "I-II" and "I-III" decreased to different degrees in 2021. Oppositely, the net input of agricultural carbon emissions from "I-I" and "I-Ⅳ" increased in 2021 compared with 2015 and 2018. Comparing with 2015 and 2018, the gap of agricultural carbon transfer became expanded among the four major net input regions of China’s agricultural carbon emissions in 2021.

**Fig 8:**
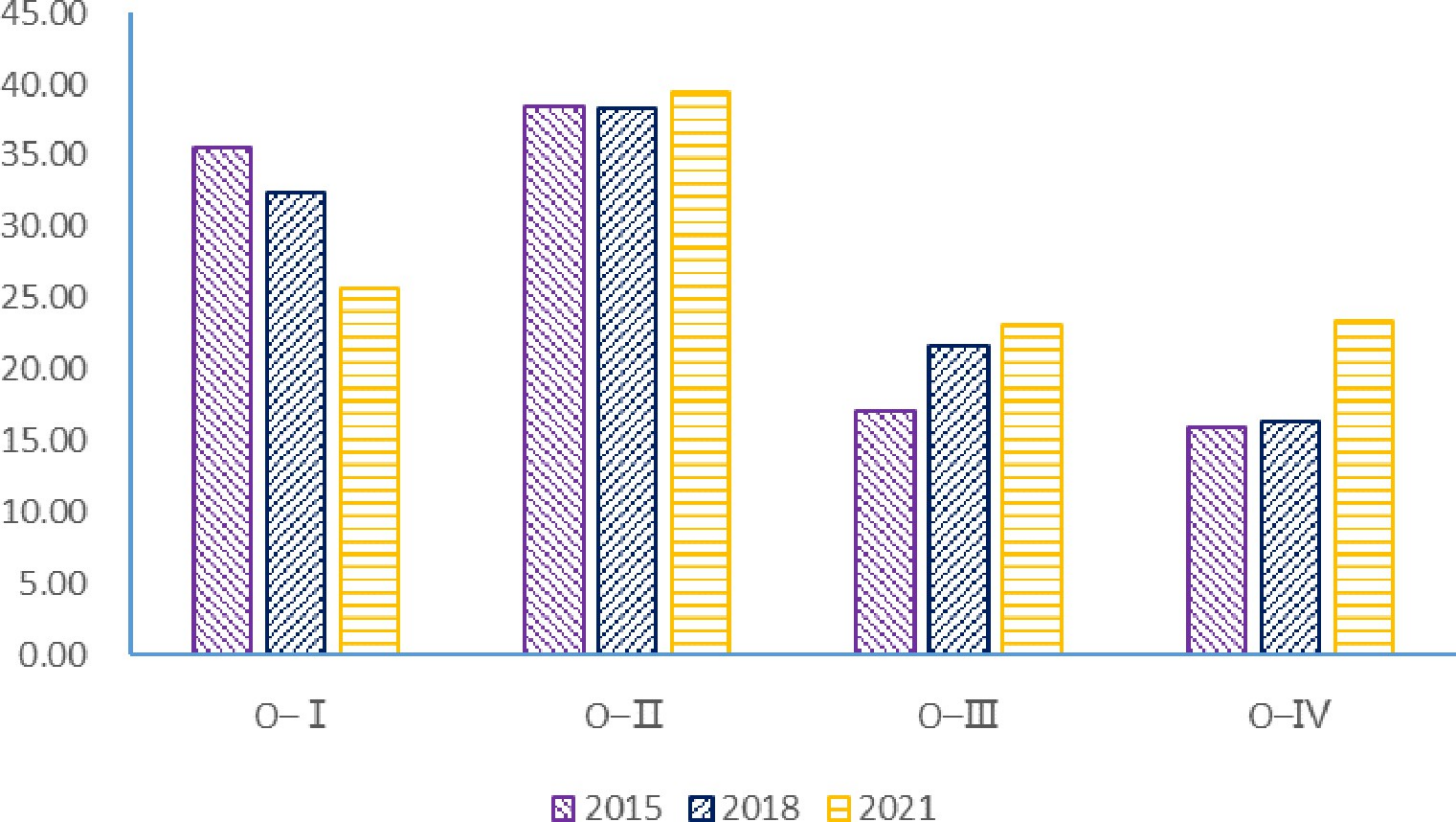
Carbon emissions from agricultural trade in major net exporting regions. (**Unit:** million tons of CE**).**

Fig 8 shows regional agricultural carbon emissions outputin 2015, 2018 and 2021, the total agricultural carbon output of "O-Ⅰ", "O-Ⅱ", "O- III " and "O-Ⅳ" regions accounted for 84.99%, 82.09% and 83.86% of the total agricultural carbon transfer, respectively, and they were the main regions exporting agricultural carbon emissions in China. Among them, the agricultural carbon emissions output in "O-Ⅰ" and "O-Ⅱ" is relatively large, while the output of "O-Ⅲ" and "O-Ⅳ" is relatively small. It is worth noting that the spatial pattern of net output of carbon emissions from agricultural trade in 2021 has changed greatly compared with before. In 2015, the scale of carbon emissions output in regions "O-Ⅰ" and "O-II" is similar, and the "O-Ⅳ" stayed the bottom. From 2015 to 2021, the net output of agricultural carbon emissions of region "O-Ⅰ" continues to decline, while the other three regions continue to rise. Overall, the net output of agricultural carbon emissions in China is still the spatial distribution pattern of the four main regions, but the center of gravity of net output is moving northward.

On the whole, the net input and output of carbon emissions from China’s agricultural trade have formed the spatial distribution pattern of four regions. Taking the Yangtze River as the boundary, the four regions with net export of agricultural carbon emissions are mainly distributed in the north of the Yangtze River, while the four regions of net input are in the south.

In order to further reflect the characteristics of interprovincial agricultural carbon emissions transfer in China, Table 5 shows the carbon transfer direction of major agricultural carbon transfer regions in China.

**Table 5:** The main flow direction of carbon emissions from China’s agricultural trade (Unit: Million tons of CE).

			Net output			
			O-Ⅳ	O-Ⅲ	O-Ⅱ	O-Ⅰ
Net input	2015	I-Ⅳ	2.77	6.46	3.29	
		I-Ⅰ	1.42		18.17	17.70
		I-Ⅱ		4.95	15.57	17.18
		I-Ⅲ	11.67	3.28	0.06	
	2018	I-Ⅳ		8.25	4.30	
		I-Ⅰ	2.59	0.94	16.27	20.13
		I-Ⅱ		5.67	14.92	12.34
		I-Ⅲ	5.26	5.69		
	2021	I-Ⅳ		10.33	3.59	
		I-Ⅰ	11.75	1.72	16.55	14.70
		I-Ⅱ		8.65	12.32	6.26
		I-Ⅲ	11.63	1.37		

The input of agricultural carbon emissions in the "I-Ⅰ" region mainly comes from the "O-Ⅱ" and "O-Ⅰ" regions. In 2015, the net agricultural carbon emissions of "O-Ⅱ" and "O-Ⅰ" to "I-Ⅰ" were 18.17million tons and 17.70 million tons, respectively, accounting for 42.25% and 41.17% of the net carbon emissions of agricultural trade in the "I-Ⅰ" region. In 2018, the net carbon emissions of "O-II" to the "I-I" area dropped to 16.27 million tons, accounting for 35.77% of the net input of "I-I" area, and the agricultural carbon emissions of "O-I" net output to "I-I" area increased to 20.13 million tons, accounting for 44.26% of the net input of "I-I" area. In 2021, the agricultural carbon emissions of "O-II" net output to "I-I" was 16.55 million tons, and the agricultural carbon emissions of "O-I" net output to "I-I" decreased to 14.70 million tons, accounting for 31.40% and 27.89% of the net carbon emissions of agricultural trade in the "I-I" region, respectively.

The sources of carbon emissions from agricultural trade in the "I-II" region are relatively dispersed, among which the "O-Ⅰ" region has the largest input. In 2015, the agricultural carbon emissions of "O-I", "O-II" and "O-III" net output to the "I-II" area were 17.18 million tons, 15.57 million tons and 4.95 million tons, respectively, accounting for 41.56%, 37.66% and 11.97% of the net carbon emissions of "I-II" agricultural trade. In 2018, the net output of agricultural carbon emissions from the "O-Ⅰ" and "O-Ⅱ" regions to the "I-II" region decreased by 4.84 million tons and 0.65 million tons, respectively, while the net output of agricultural trade carbon emissions from the "O-Ⅲ" region to the "I-II" region increased to 0.72 million tons. By 2021, the net output of agricultural carbon emissions from the "O-Ⅰ" and "O-Ⅱ" regions to the "I-II" region further decreased by 10.92 million tons and 3.25 million tons, respectively, while the net output of agricultural carbon emissions from the "O-Ⅲ" region to the "I-II" region increased by 3.70 million tons. On the whole, the agricultural carbon emissions output from the "O-Ⅰ" and "O-II" regions to the "I-II" region became decreased while the agricultural carbon emissions output from the "O-Ⅲ" region to the "I-II" region increased.

The net input of agricultural carbon emissions in the "I-Ⅲ" region mainly comes from the "O-Ⅳ" and "O-Ⅲ" regions. In 2015, the net agricultural carbon emissions from the "O-IV" and "O-III" regions to the "I-III" regions were 11.67 million tons and 3.28 million tons, accounting for 50.80% and 14.28% of the net carbon emissions from the "I-III" agricultural trade, respectively. In 2018, the net agricultural carbon emissions from the "O-Ⅲ" region to the "I-Ⅲ" increased by 2.41 million tons while the agricultural carbon emissions output from the "O-Ⅳ" region to the "I-Ⅲ" region decreased. In 2018, agricultural carbon emissions in "I-Ⅲ" mainly came from the "O-Ⅳ" and "O-Ⅲ", which accounts for 42.36% and 45.82% of its emissions respectively.

In 2021, the net agricultural carbon emissions from the "O-IV" and "O-Ⅲ" to the "I-III" decreased by 0.05 million tons and 1.91 million tons, respectively. It is worth noting that in 2021, agricultural carbon emissions from "O-IV" to "I-III" accounted for 81.90% of its input carbon emissions, making "O-IV" the most important source of agricultural carbon emissions in "I-III". The net input of agricultural carbon emissions in "I-Ⅳ" region mainly comes from "O-Ⅱ" and "O-Ⅲ". In 2015 and 2018, the net output of agricultural carbon emissions to the "I-Ⅳ" region was mainly the "O-Ⅲ" region, and the carbon emissions of agricultural trade were 6.46 million tons and 8.25 million tons, respectively, accounting for 51.60% and 65.75% of the net input of agricultural carbon emissions in the "I-Ⅳ" region, respectively. In 2021, the agricultural carbon emissions of "O-III" net output to the "I-IV" region rised to 10.33 million tons, accounting for 74.20% of the net agricultural carbon emissions input of "I-IV" region. It can be seen that the increase in the net input of agricultural carbon emissions in the "I-IV" region is mainly due to the inflow of carbon emissions in the "O-III" region. On the whole, agricultural carbon emissions transfer present to flow between the north and the south to more things to the characteristics of less. In 2015, 2018 and 2021, China’s agricultural carbon transfer showed obvious characteristics of "north carbon transport to south". Among them, the middle flow is from north to south, the bilateral flow is from northwest to southwest, and the northeast to southeast.

Table 5 shows the quantity and ranking of agricultural carbon emissions transfer in China. From the perspective of the changes in the ranking of agricultural carbon transfer volume, the size and ranking of carbon transfer volume from net agricultural carbon emissions output to net agricultural carbon emission input regions in 2015 and 2018 are relatively similar, and great changes have taken place in 2021. In 2015, 2018, China’s agricultural products trade in carbon emissions in the top four for "O-Ⅰ" output to the "I-Ⅰ", "O-Ⅰ" output to the "I-Ⅱ", "O-Ⅱ" output to the "I-Ⅱ" and "O-Ⅱ" output to the "I-Ⅰ". In 2021, China’s agricultural carbon migration in the top four for "O-Ⅱ" output to the "I-Ⅰ", "O-Ⅰ" output to the "I-Ⅰ", "O-Ⅱ" output to the "I-Ⅱ" and "O-Ⅳ" regional output to the "I-Ⅰ" area. The above analysis shows that the agricultural carbon emissions from "O-Ⅰ" region decreased more, while the agricultural carbon emissions from "O-Ⅳ" region increased more. The carbon emissions of agricultural trade from "O-IV" net output to "I-Ⅰ" in 2021 also increased significantly compared with 2015 and 2018. "O-Ⅳ" from the main output in 2015 to "I-III", to 2018 the main net output to "I-III", small-scale net output to "I-Ⅰ", and then to 2021 with net output to "I-Ⅰ" and "I-III" mainly, to achieve a rapid growth in the net output of agricultural carbon emissions. In this process, not only the net output of carbon emissions from "O-I" to "I-II" and "I-I" agricultural trade is squeezed. In addition, compared to 2015 and 2018, the carbon emissions of agricultural products exported from "O-III" to "I-IV" in 2021 also increased.

Based on the aforementioned study, we have learned that the net carbon transfer in Chinese agriculture mainly occurs between eight regions. In 2015, 2018, and 2021, the net carbon transfer volume of these eight regions was 102.54 million tons, 96.35 million tons, and 98.86 million tons, respectively, accounting for 81.53%, 72.73%, and 74.32% of the total net carbon transfer volume. Does this mean that the agricultural carbon transfer flow in the remaining areas is relatively low? To answer this question, we further distinguish the total net agricultural carbon transfer into two categories: implicit carbon transfer of grain and carbon transfer of livestock and poultry breeding, as shown in Tables 6 and 7, respectively.

**Table 6:** Carbon emissions from grain (Unit: Million tons of CE).

			Net output				
			O–Ⅰ	O–Ⅱ	O–Ⅲ	O–Ⅳ	O-R
Net input	2015	I–Ⅰ	9.16	8.52			5.05
		I–Ⅱ	4.09	7.81	4.95		1.55
		I–Ⅲ			1.72	6.58	1.74
		I–Ⅳ		3.09			
		I–R	6.41	9.18	1.88	4.19	
	2018	I–Ⅰ	12.03	9.53			4.70
		I–Ⅱ	7.64	7.10	5.67		
		I–Ⅲ			3.70	6.17	
		I–Ⅳ		2.58			
		I–R	3.77	10.67	0.39	2.48	2.22
	2021	I–Ⅰ	8.31	10.94			4.64
		I–Ⅱ	6.97	10.75	0.41		
		I–Ⅲ			3.05	5.83	1.22
		I–Ⅳ		1.83			
		I–R	5.32	6.05	7.54	3.74	

**Table 7:** Carbon emissions from livestock and poultry breeding (Unit: Million tons of CE).

			Net output				
			O–Ⅰ	O–Ⅱ	O–Ⅲ	O–Ⅳ	O-R
Net input	2015	I–Ⅰ	2.92	9.65			7.71
		I–Ⅱ	12.01				10.93
		I–Ⅲ				5.10	7.85
		I–Ⅳ		0.21	6.46		2.77
		I–R	0.95		2.02		5.77
	2018	I–Ⅰ	4.33	6.66	0.94		7.31
		I–Ⅱ	4.70				16.09
		I–Ⅲ			1.87	7.66	9.60
		I–Ⅳ		1.72	8.25		
		I–R			0.92		6.38
	2021	I–Ⅰ	5.25	1.30	1.71	6.88	13.66
		I–Ⅱ					18.42
		I–Ⅲ				6.75	0.28
		I–Ⅳ		1.76	10.33		
		I–R		6.81		0.17	7.44

Looking at the grain carbon transfer situation, from 2015 to 2018 to 2021, the net carbon transfer volume of the eight main regions was 45.91 million tons, 54.42 million tons, and 48.09 million tons, respectively, accounting for 60.48%, 69.19%, and 62.77% of the grain net carbon transfer volume. The grain trade carbon transfer shows a spatial transfer characteristic from the northern regions to the southeastern coastal areas as the main direction, with the southwestern regions as a secondary direction. Regarding the carbon transfer from livestock and poultry breeding, the net carbon transfer volume of the eight main regions from 2015 to 2018 to 2021 was 36.35 million tons, 36.11 million tons, and 33.99 million tons, respectively, accounting for 48.90%, 47.26%, and 42.08% of the net carbon emissions from livestock and poultry breeding. The carbon transfer of meat products indicates a main characteristic of transferring from the north to the south, with a secondary direction from the southwest to the southeast. The results show that the net agricultural carbon transfer of the eight regions is much higher than their grain net carbon transfer and the net carbon transfer from livestock and poultry breeding, which means that the agricultural carbon transfer flow in the remaining areas is not low but plays an intermediary role in the agricultural carbon transfer process. Taking Yunnan in 2021 as an example, it is a province with a high net input of implicit carbon from grain trade and also a province with a high net output of implicit carbon from meat products. Therefore, the net effect is that Yunnan is a province with agricultural trade implicit carbon emissions. The main reason is that Yunnan has a large gap in grain demand, and the implicit carbon emissions from net imported grain trade offset the implicit carbon emissions from net exported meat product trade to some extent.

## Discussion

This part analyzes the causes of agricultural carbon transfer in China, and discusses the reasons for the changes in the characteristics of agricultural carbon transfer in recent years. In addition, it also presents the limitations of this study.

### Main results and causes of agricultural carbon transfer direction

From the perspective of spatial analysis, the pattern of agricultural carbon emissions input and output in China is mainly divided by the Yangtze River. The output regions are mainly concentrated in the north of the Yangtze River, while the input ones are mainly located in the south. Such distribution shows a spatial carbon transfer feature of "north carbon transport to south".

In recent years, many scholars have carried out a lot of research on China’s inter-regional agricultural trade focusing on the virtual water transfer embedded in grain. Studies have shown that virtual water embedded in food has moved from the water-scarce northern region to the water-rich southern region, and it increased from 7.299 billion cubic meters in 1997 to 12.464 billion cubic meters in 2014 [43]. Some scholars also used linear programming model to carry out research, and the results showed that from 2004 to 2013, the virtual water flow of grain in China mainly shifted from the north to the south [67]. There are also other studies on the virtual water flow characteristics of China’s grain, and the results indicate that the virtual water flow of China’s grain presents the characteristics of "water moving from north to south" [46, 68, 69]. Some scholars have also studied the transfer of carbon emissions from China’s grain trade, which also supports the result of "North carbon transport to south" [38]. In this paper, it not only considers the regional trade of grain but also the regional trade of meat products to avoid ignoring the carbon transfer of meat when. Upon the comprehensive consideration of carbon transfer in agricultural trade, this paper finds that the "carbon transport from north to south" is the main feature of carbon transfer in China’s agriculture.

There are four main aspects. First, the acceleration of urbanization in southern China led to an increase in food demand, while the agricultural output in southern China decreased after the conversion of agricultural land to industrial land [70], which led to the imbalance between production capacity and consumption level. Second, differences in economic development between regions have widened. The more developed the regional economy, the higher the demand for agricultural products. China’s five major urban agglomerations are Yangtze River Delta, Pearl River Delta, Chengdu-Chongqing, Beijing-Tianjin-Hebei and the middle reaches of the Yangtze River [71]. These major urban agglomerations are regions higher economic development places in China. This is highly consistent with the regions of agricultural carbon input in China concluded in this paper. The third one is the differences of agricultural resource endowments. China’s agricultural carbon output area is mainly concentrated in the region north of the Yangtze River, mainly because the population in northern China is relatively small and the agricultural production conditions are better. In 2019, the per capita cultivated land area in China was 0.09 ha/person, and the per capita cultivated land area in Heilongjiang, Inner Mongolia, Jilin, Xinjiang and Gansu was 0.53 ha/person, 0.48 ha/person, 0.31 ha/person, 0.28 ha/person and 0.21 ha/person respectively, and these numbers were far higher than the one of national average and other provinces. As a result, agricultural output produced in these regions far exceeds the amount of local consumption demand. Fourth, the factor of population mobility. From the perspective of inter-provincial population mobility, the regions with a net outflow of population are mainly northern provinces such as Henan, Jilin, and Heilongjiang, while the regions with a net inflow of population are mainly Guangdong, Zhejiang, Sichuan, and Chongqing. The majority of migrants tend to move from the northern regions to the eastern coastal urban clusters and inland provincial capitals [72]. The direction of population mobility shows a certain correlation with the direction of agricultural carbon transfer.

### Analysis of the reasons why the center of gravity of agricultural carbon transfer is shifting to the north

From the perspective of time, the center of gravity of agricultural carbon emissions transfer is changing in China, and the most important feature of the change is the decline of carbon emissions output in "O-Ⅰ" region and the increase of carbon emission output in northern region. Below, we analyzed the reasons for such structural changes in agricultural carbon transfer output.

**Fig 9:**
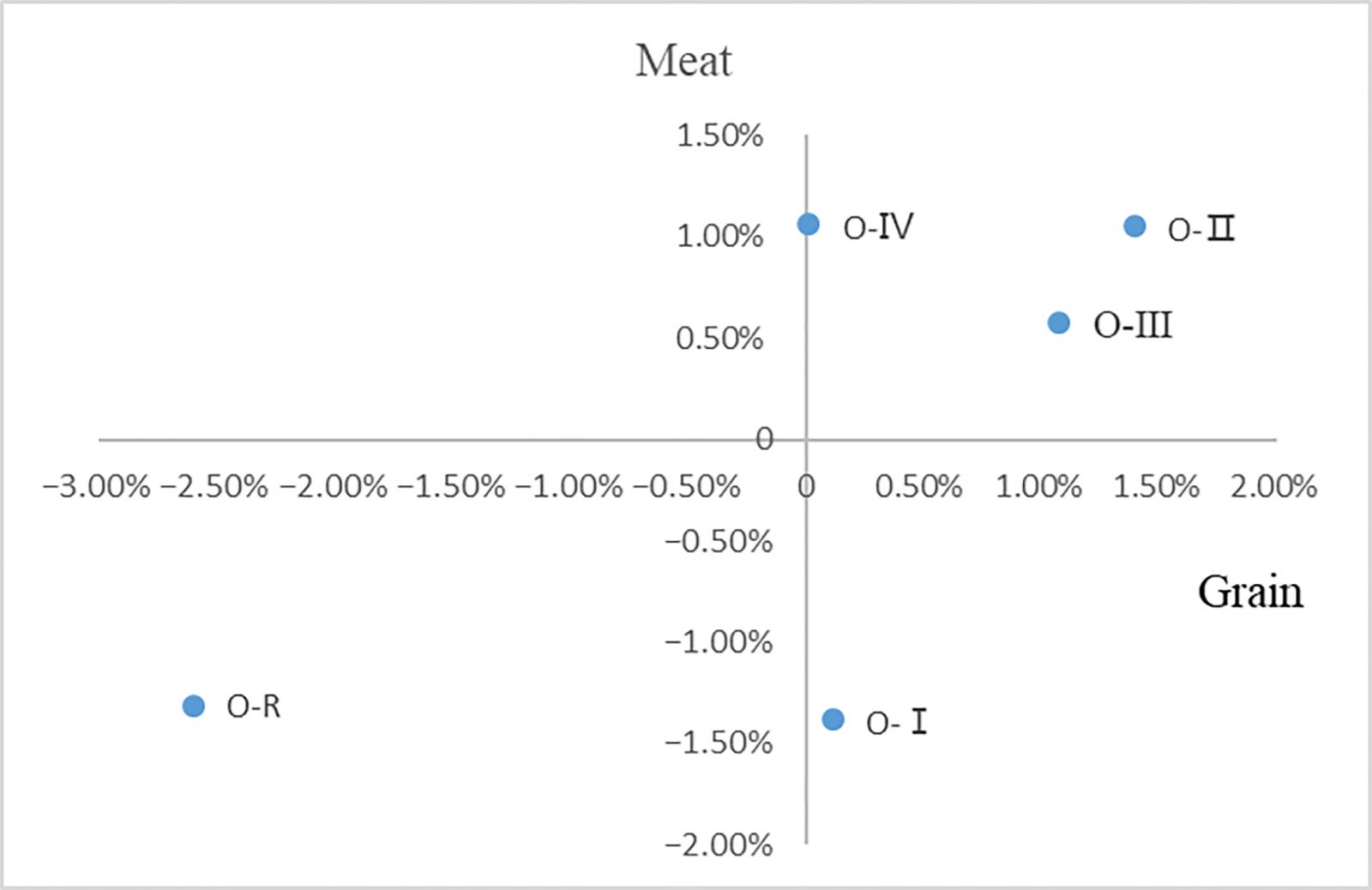
Relative changes of regional agricultural product supply in 2015 and 2021.

**Fig 10:**
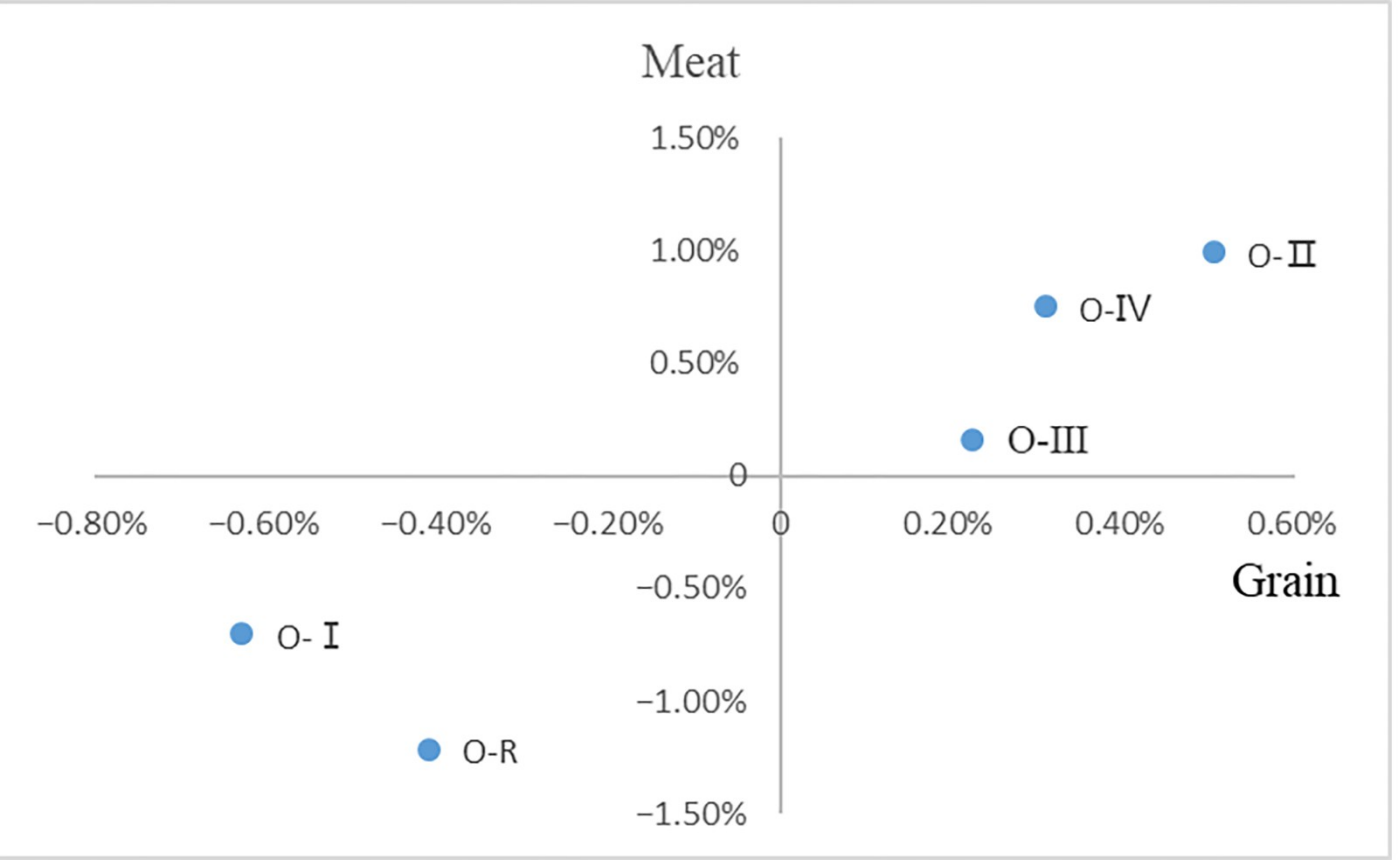
Relative changes of regional agricultural products supply in 2018 and 2021.

First, the northern region has increased its deliverability to the whole country. This paper calculates and compares the changes in the supply of agricultural products in major regions in 2021, 2015 and 2018 respectively, as shown in Figs 9 and 10. Compared with 2015, the proportion of grain production in the "O-I" region in 2021 increased by 0.11% of the total national output, and its deliverability to the country was enhanced, while the proportion of meat production in the total national output fell by1.38%, and the deliverability to the country declined. At the same time, "O-Ⅳ", "O-Ⅱ" and "O-Ⅲ" regions showed an increasing trend in the proportion of grain production and meat production. Compared with 2018, the proportion of grain production and meat production in the "O-Ⅰ" region in 2021 showed a significant decline, down 0.63% and 0.70% respectively, and the supply capacity of the country declined. At the same time, the proportion of grain and meat production in the "O-Ⅳ", "O-Ⅲ" and "O-Ⅱ" regions has increased. Therefore, the overall output of agricultural products in "O-Ⅳ", "O-Ⅱ" and "O-Ⅲ" increased in 2021 comparing to 2015 and 2018, while the output of agricultural products in "O-Ⅰ" and other regions decreased, it resulted in product supply shifting to the north in the agricultural trade.

**Fig 11:**
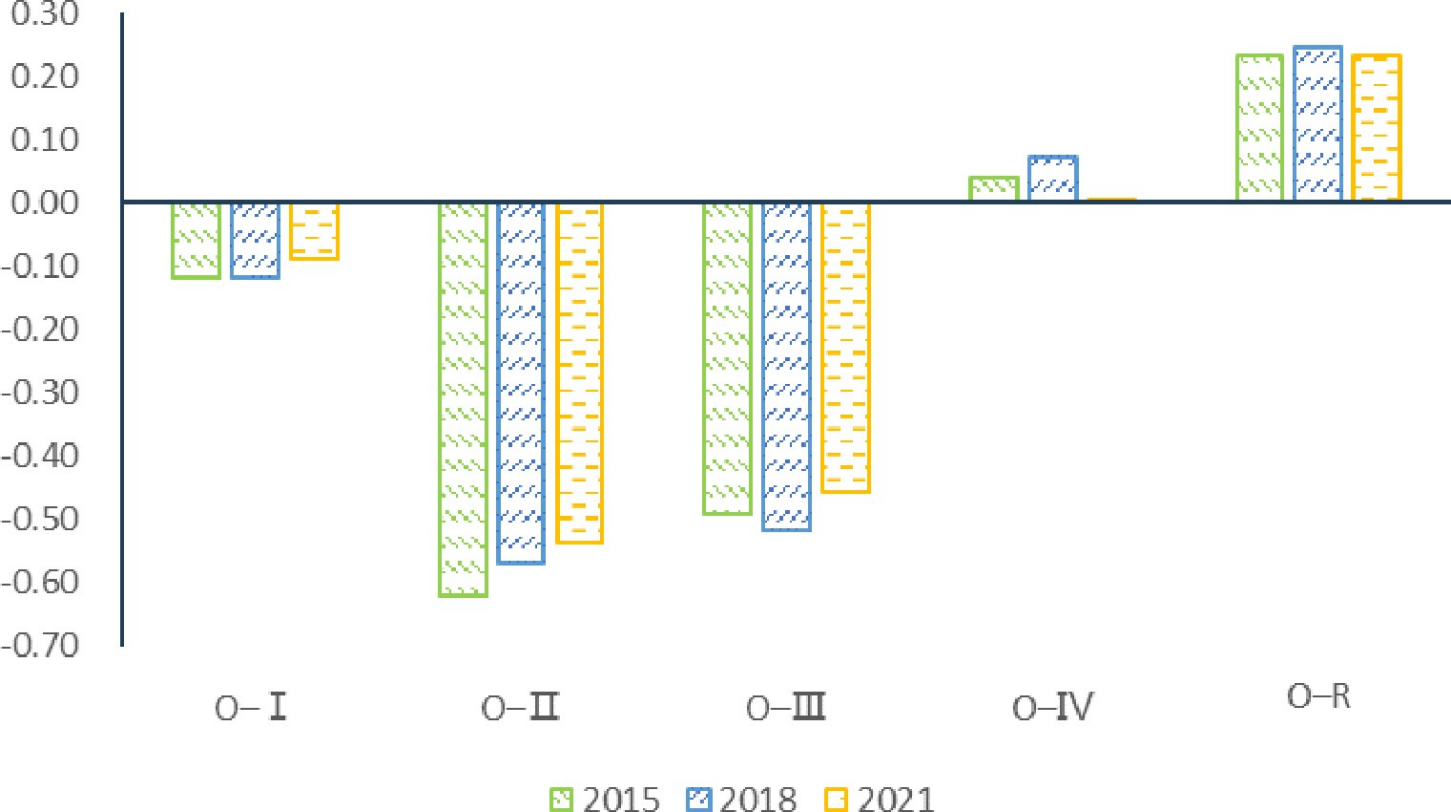
Relative coefficient of grain carbon emission. (**Unit:** CE/t**).**

**Fig 12:**
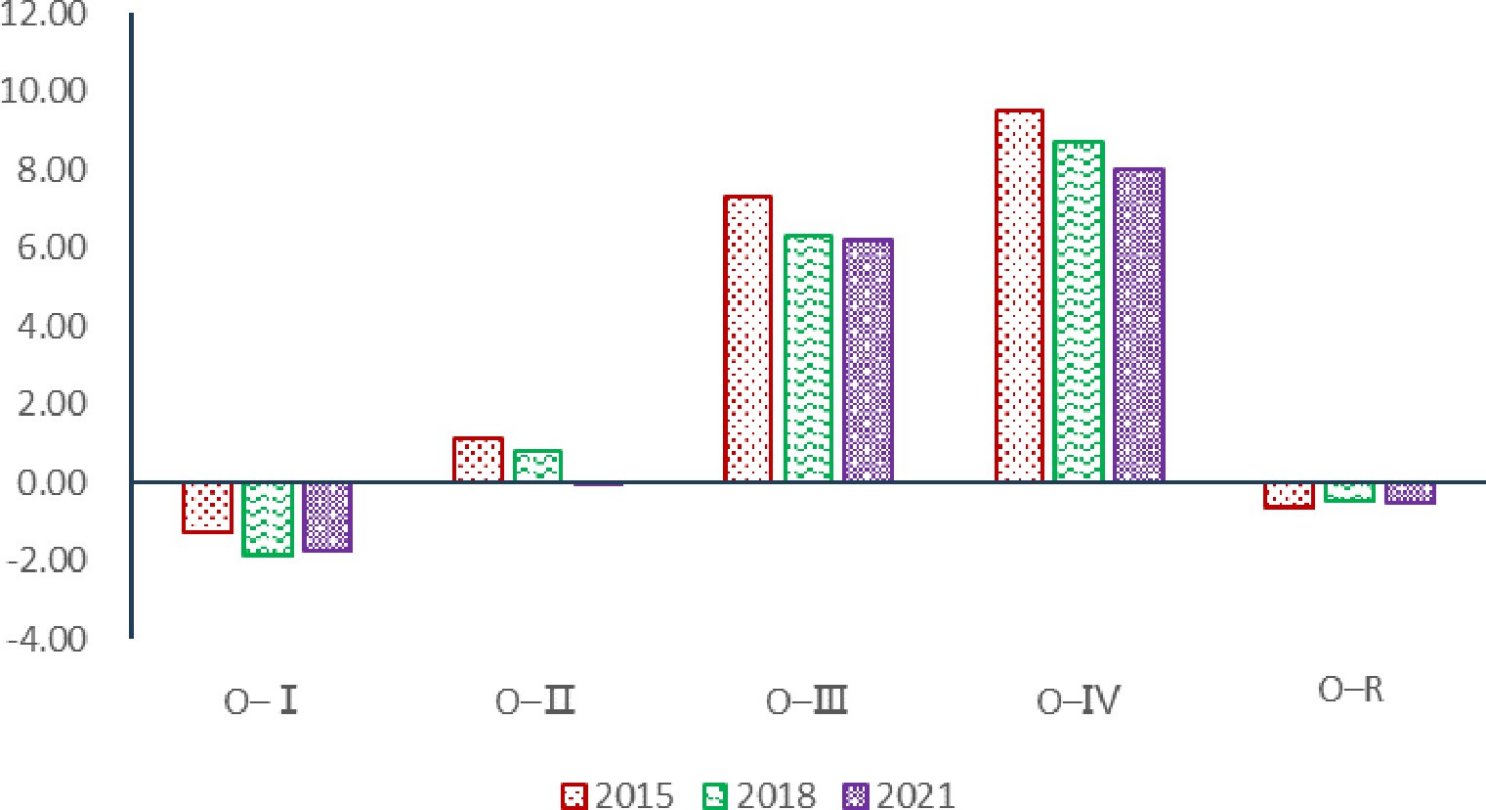
Relative coefficient of meat carbon emission. (**Unit:** CE/t**).**

Second, northern China has lower level of low carbon development in livestock and poultry farming. This paper calculates the relative carbon emission coefficient of agricultural products in 2015, 2018 and 2021 and uses the difference between regional and the national average carbon emission coefficient of agricultural products as an indicator to measure the low carbon level of agricultural production in each region. Figs 11 and 12 show the relative carbon emission coefficients of grain and meat production respectively. Fig 11 shows that from the perspective of grain production, the carbon emission coefficient of grain production in "O-Ⅲ", "O-Ⅱ" and "O-Ⅰ" regions is lower than the national average, indicating that the low carbon production level of grain production in these three regions is relatively high. While the carbon emission coefficient of grain production in "O-Ⅳ" region is higher than the national average, and it means the "O-Ⅳ" region is the worst among the four food-carbon emitting regions in low carbon production level of grain production.

Fig 12 shows the carbon emission coefficient of meat production in "O-Ⅲ", "O-Ⅳ" and "O-Ⅱ" regions is higher than the national average from the perspective of meat production, indicating that the low-carbon production level of meat production in these three regions is relatively low. The carbon coefficient of meat production in the "O-Ⅰ" region is lower than the national average. From the point of view of meat production, the "O-Ⅰ" region ranks first among the four meat carbon emission regions in the low carbon level of meat production. Therefore, the "O-Ⅰ" region enjoys the lowest the carbon emission coefficient of meat production in the development process of livestock and poultry industry, and its low-carbon development level is higher than that of the other three main output regions. It is the poor level of low-carbon development of livestock and poultry industry in the "O-Ⅱ", "O-Ⅲ" and "O-Ⅳ" regions that further leads to the northward shift of the gravity center of carbon emission export in agricultural trade.

### Comparison of results

At present, some studies have utilized multi-regional input-output analysis to examine the issue of implicit resource flows in agriculture, such as research on the agricultural water footprint (AWF). Ren et al. used the multi-regional input-output table from 2007 to investigate the issue of virtual water flows in agriculture within the Jing-Jin-Ji region. Their study found that Beijing and Tianjin are regions where virtual water from agriculture flows in, while Hebei is a region where it flows out. The results of this paper also show that Beijing and Tianjin are regions of agricultural carbon emission input, and Hebei is a region of agricultural carbon emission output [42]. Sun et al. employed multi-regional input-output analysis to compute the agricultural water, land, and carbon footprints across 30 provincial-level administrative regions in China. The findings indicate that these footprints predominantly shift from the western and northern parts of the country towards the eastern and southeastern regions, demonstrating that the consumption of agricultural products in the eastern and southeastern areas has resulted in the virtual appropriation of water and land resources and the embedded carbon emissions in the western and northern areas. Regions with a net outflow of virtual water are primarily located in the northern Xinjiang and Heilongjiang, whereas those with a net inflow are predominantly found along the coast in provinces such as Guangdong, Jiangsu, and Zhejiang [73]. Ju et al. used the multi-regional input-output method to investigate the flow of inter-provincial agricultural water footprint (AWF) in China, discovering that the agricultural water footprint is generally higher in the northern regions, with the virtual water mainly shifting from the northern areas to the coastal regions [74]. Bai et al. combined the Environmentally Extended Multi-Regional Input-Output (EEMRIO) and Structural Decomposition Analysis (SDA) approaches to examine the transfers of virtual water, land, and carbon emissions within China’s regional trade. The findings reveal that between 2007 and 2017, the implicit carbon emissions from agriculture predominantly moved from the northern regions of China to the southern and southeastern regions. Xinjiang and Heilongjiang are the provinces with significant outflows of virtual water, whereas Guangdong, Zhejiang, and Shanghai are the provinces with notable inflows of virtual water [75]. Tong et al. utilized spacetime clustering analysis and coupled analysis of virtual water-economic and social data to elucidate the significant spatial clustering of grain virtual water transportation. Their research examined the spatiotemporal patterns of virtual water flow across 31 Chinese provinces from 1997 to 2021. The findings indicate that the general flow of agricultural virtual water originates from the northern regions and moves towards the southern regions, as well as from areas with less economic development to those that are more economically advanced. Heilongjiang, Jilin, and Inner Mongolia are identified as the primary regions of agricultural virtual water outflow, whereas Zhejiang, Guangdong, Shanghai, and Fujian are recognized as the principal regions of agricultural virtual water inflow [76].

The aforementioned research findings utilizing the multi-regional input-output approach exhibit some minor discrepancies in specific details when compared to this paper, but they collectively endorse the research conclusion presented here, which is that the overall spatial transfer characteristic of China’s agricultural carbon transfer demonstrates a "north carbon transport to south" pattern.

### Limitation

The limitations of this paper are reflected in two aspects. On the one hand, when calculating the inter-provincial trade of agricultural products, it is first necessary to construct a balance sheet for agricultural products. Due to the lack of consumption data for agricultural products by urban and rural residents by province before 2015, there are difficulties in constructing the balance sheet for agricultural products. Therefore, it fails to reflect the comprehensive trend of changes in carbon transfer. On the another hand, we use a linear programming model to simulate the trade of agricultural products. It is assumed that there is no difference in the production of similar agricultural products in different provinces, yet in fact, there is a certain degree of heterogeneity in the production of similar agricultural products in different provinces. Although food and meat production can represent most of the carbon emissions from farming and livestock production, there is still some margin of errors. For example, besides grain production, planting industry also produces vegetables and fruits. Livestock and poultry industry not only produces meat but also eggs and milk. In the future, we will dedicate to model improvement and make more realistic research results.

## Conclusions and policy implications

### Conclusions

Based on the calculation of agricultural carbon emissions in China, this paper analyzes the spatio-temporal evolution of agricultural carbon transfer in China. The results show that the carbon emissions input area of China’s agriculture is mainly located in the south of the Yangtze River, while the output area is mainly located in the north. Specifically, the input areas of agricultural carbon emissions in China formed a spatial distribution pattern of four major regions with the input gravity center of the southeast coastal zone, and the gap between the input amounts of carbon emissions among the four major regions was widening, and the phenomenon of "polarization" was becoming more and more obvious. China’s agricultural carbon emissions output area also formed a spatial distribution pattern of four major regions with the output center of gravity being the northern provinces near the border, and the output center of gravity was moving northward. On the whole, China’s agricultural carbon transfer shows an obvious feature of "north carbon transport to south". As time goes by, the output of agricultural carbon emissions in of the output region "O-Ⅰ" is declining, while it rises in "O-Ⅱ", "O-Ⅲ" and "O-Ⅳ" regions.

### Policy implications

First, it suggests increasing the self-sufficiency of agricultural products in carbon-emitting provinces, such as Beijing, Tianjin, and Shanghai, which have scarce agricultural land but a high level of economic development, should further accelerate the development of facility agriculture, construct modern agricultural factories, and enhance the high-end, intelligent, and large-scale production of agriculture to improve local supply capacity. For areas like Sichuan, Chongqing, Guizhou, Guangdong, Guangxi, and Fujian, it is necessary to strengthen farmland management to prevent "non-agricultural" and "non-grain" use of farmland, increase efforts in agricultural talent cultivation, enhance the supply of agricultural labor, promote agricultural land use in hilly and mountainous areas, construct high-standard farmland, and develop multi-season grain planting according to local conditions. Additionally, strengthen the management of the livestock and poultry breeding industry, promote the construction of large-scale breeding farms, and improve the management of livestock and poultry breeding.

Second, it shall focus on improving the efficiency of green and low-carbon agricultural production. Important grain-producing areas such as Heilongjiang, Jilin, Henan, and Anhui, which mainly export grain carbon emissions, need to continue to solidify their role in grain production. Deepen the protection and improvement of farmland quality, improve the mechanism for food security and interest compensation, increase the income from grain planting, motivate farmers’ enthusiasm for production, and maintain stable grain production capacity. It is especially important to focus on improving planting technology, reducing the use of pesticides and fertilizers, optimizing the allocation of water and soil resources, and improving the production efficiency of grain crops. Regions with large agricultural carbon emissions such as Inner Mongolia, Xinjiang, Gansu, Yunnan, Qinghai, and Tibet, which are also net exporters of agricultural carbon emissions and mainly export carbon emissions from livestock and poultry breeding, need to strengthen grain production and improve the management of the livestock and poultry breeding industry. Reduce small-scale and scattered breeding enterprises, promote large-scale and intensive management of livestock and poultry breeding.

Third, stablish inter-provincial agricultural cooperation mechanisms. The net input regions of agricultural carbon emissions are mostly concentrated in the economically developed areas along the southeast coast, while the net export regions of agricultural carbon emissions are mainly in the northern and western regions of China. The economically developed areas along the southeast coast have relatively high agricultural green and low-carbon efficiency, so it is possible to build a cooperative mechanism for agricultural production between regions. Strengthen cooperation in crop planting technology, livestock and poultry breeding management, and agricultural production talents to further enhance agricultural green and low-carbon efficiency.

Fourth, it suggests exploring pilot carbon offsets based on the transfer of carbon emissions from agricultural trade. The existing agricultural interest compensation is mainly a vertical compensation from the central government to local areas. In recent years, the state has issued documents many times to establish an inter-provincial horizontal agricultural interest compensation mechanism. However, due to difficulties in compensation scope, compensation methods, and compensation standards, a feasible policy system has not yet been formed. The agricultural carbon transfer issues studied in this paper can provide ideas for constructing an inter-provincial horizontal agricultural carbon compensation mechanism. It can refer to the price standards of the national carbon market, take the implicit carbon transfer amount of inter-provincial agricultural products as the basis, clarify the net carbon transfer direction between provinces, and then determine the amount of inter-provincial horizontal agricultural carbon compensation.

In different countries, especially major agricultural countries, there is a certain degree of agricultural carbon transfer due to differences in regional economic development and population mobility. The research in this paper provides reference significance for agricultural carbon emission reduction in similar countries around the world. Firstly, attention should be paid to improving the level of agricultural green and low-carbon efficiency to reduce agricultural carbon emissions in the production process of agricultural products. Secondly, the self-sufficiency capacity of agricultural products should be enhanced to increase the supply of local agricultural products and reduce additional carbon emissions caused by the transportation of agricultural products. Thirdly, from a global perspective, developed regions should increase support for the agricultural development of less developed areas, increase financial investment, technical support, and personnel training, to enhance the output of agricultural products in less developed areas and reduce the carbon emissions per unit of agricultural product produced.

## Supporting information

S1 Fig Interprovincial agricultural carbon emission and carbon transfer in 2015. (XLSX)

S2 Fig Interprovincial agricultural carbon emission and carbon transfer in 2018. (XLSX)

S3 Fig Interprovincial agricultural carbon emission and carbon transfer in 2021. (XLSX)

S4 Fig Carbon emissions flows in agricultural trade in 2015. (XLS)

S5 Fig Carbon emissions flows in agricultural trade in 2018. (XLS)

S6 Fig Carbon emissions flows in agricultural trade in 2021. (XLS)

S7 Fig Carbon emissions from agricultural trade in major net importing regions. (XLSX)

S8 Fig Carbon emissions from agricultural trade in major net exporting regions. (XLSX)

S9 Fig Relative changes of regional agricultural product supply in 2015 and 2021. (XLSX)

S10 Fig Relative changes of regional agricultural product supply in 2018 and 2021. (XLSX)

S11 Fig Relative coefficient of grain carbon emission. (XLSX)

S12 Fig Relative coefficient of meat carbon emission. (XLSX)

S1 Table Agricultural carbon emission volumes of different provinces in 2021. (XLSX)

S2 Table Proportion of agricultural carbon transfer in different provinces. (XLSX)

S3 Table The main flow direction of carbon emissions from China’s agricultural trade. (XLS)

S4 Table Carbon emissions from grain. (XLS)

S5 Table Carbon emissions from livestock and poultry breeding. (XLS)
